# Portable device for dual detection of fluorescence and absorbance for biosensing or chemical sensing applications

**DOI:** 10.1016/j.ohx.2022.e00268

**Published:** 2022-01-28

**Authors:** Kittirat Phooplub, Sirirat Ouiganon, Panote Thavarungkul, Proespichaya Kanatharana, Chittanon Buranachai

**Affiliations:** a Division of Physical Science, Faculty of Science, Prince of Songkla University, Songkhla, Thailand; b Thailand Center of Excellence in Physics, Commission on Higher Education, 328 Si Ayutthaya Road, Bangkok, Thailand; c Center of Excellence for Trace Analysis and Biosensor, Faculty of Science, Prince of Songkla University, Songkhla, Thailand; d Center of Excellence for Innovation in Chemistry, Faculty of Science, Prince of Songkla University, Songkhla, Thailand

**Keywords:** Portable fluorometer, Portable spectrophotometer, Biosensor, Chemical sensor, On-site detection

## Abstract

Early detection is key to prevent health problems and could be performed by biosensors and chemical sensors. However, a lot of them still need bulky benchtop equipment. This work presents a portable device for measuring fluorescence and light absorption that can be used with optical biosensors or chemical sensors. It uses a small laser diode as a light source and three filter-mounted photodiodes as detectors, all of which are inexpensive, customizable and widely available commercially. The results from our device show good correlation with that from commercial instruments. Therefore, it could be beneficial for early or on-site detection.

## Highlights

• A portable device for dual detection of fluorescence and light absorption.
• It is inexpensive, customizable and easy to operate with minimal maintenance.
• Results are in good correlation with that form commercial instruments and requires only 500 μl of sample volume.

## Graphical abstract

**Figure:**
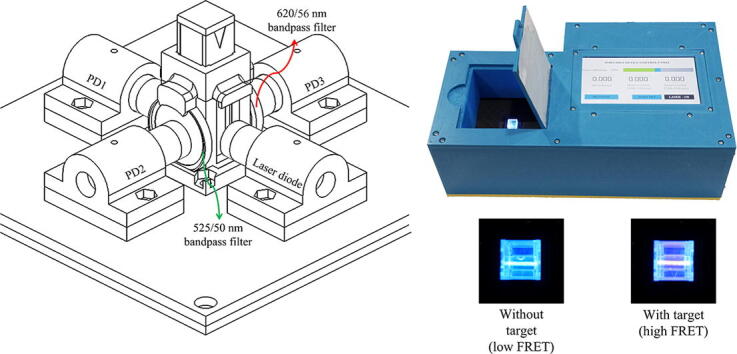

## Specifications table

**Table:** 

Hardware name	*Portable device for dual detection of fluorescence and absorbance for biosensing or chemical sensing applications*
Subject area	• Optical Biosensors • Chemical Sensors • Early Diagnosis
Hardware type	• Field measurements and sensors • Other: low cost alternatives to existing tools
Closest commercial analog	• Fluorometer (e.g., F-2700, Hitachi, Japan) • Absorption spectrophotometer (e.g., U-2900, Hitachi, Japan) • Portable fluorometers (e.g., Qubit Fluorometers, Thermo Fisher Scientific, USA)
Open source license	Creative Commons Attribution 4.0 International (CC BY 4.0)
Cost of hardware	USD 1,100
Source file repository	Mendeley Data https://doi.org/10.17632/bdg2c7tsxm.1

## Hardware in context

Early detection of biomarkers in the bodies or the level of toxic chemicals or pyrogens in foods is an important preventive measure to tackle health problems. However, most of the analyses are performed by highly skilled personal in well-equipped laboratories, which are not easily accessible, especially in remote areas or in developing countries. An interesting alternative is to use biosensors or chemical sensors that offer high selectivity, high sensitivity, low detection limit, ease of use and, quite often, rapid detection compared with conventional methods in laboratories. Despite all of the benefits, a lot of them still rely on benchtop equipment in laboratories. For examples, there are various optical bio- and chemical sensors developed for the detection of key biomarkers or analytes that are important in healthcare and food safety applications. Most of them uses either changes in sensors’ fluorescence emission or light absorption to signal the binding and a lot of them still rely on benchtop fluorometers or absorption spectrometer to measure the signals.

To address the problem, several research groups introduced portable fluorescence detectors or absorption spectrometers that can be used outside laboratories [1], [2], [3], [4]. In fact, some of them are commercially available, such as Qubit Fluorometers (Fisher Scientific, USA) or Optizen mini UV–Vis Spectrophotometer (K LAB, Republic of Korea). However, they require teams of experts to develop and the commercial models are still expensive for most of the developing countries. In addition, to the best of our knowledge, none of them is capable of measuring both fluorescence emission and light absorption. In this work, we provide detailed instructions, lists of materials and downloadable STL (stereolithography) and g-code files for constructing a portable device for dual detection of fluorescence and light absorption. It is in our best interest that it should widely reach audience being interested in on-site or point-of-care applications, especially in areas with limited resources. However, it must be emphasized that we do not intend to replace commercial benchtop fluorometers and UV–Vis absorption spectrometers with our device; it is good for applications that has a clear target and do not require spectral measurement.

## Hardware description

The device was developed based on opensource technology as follow:

### Fluorometer and absorption spectrometer setup

**Fig. 1:**
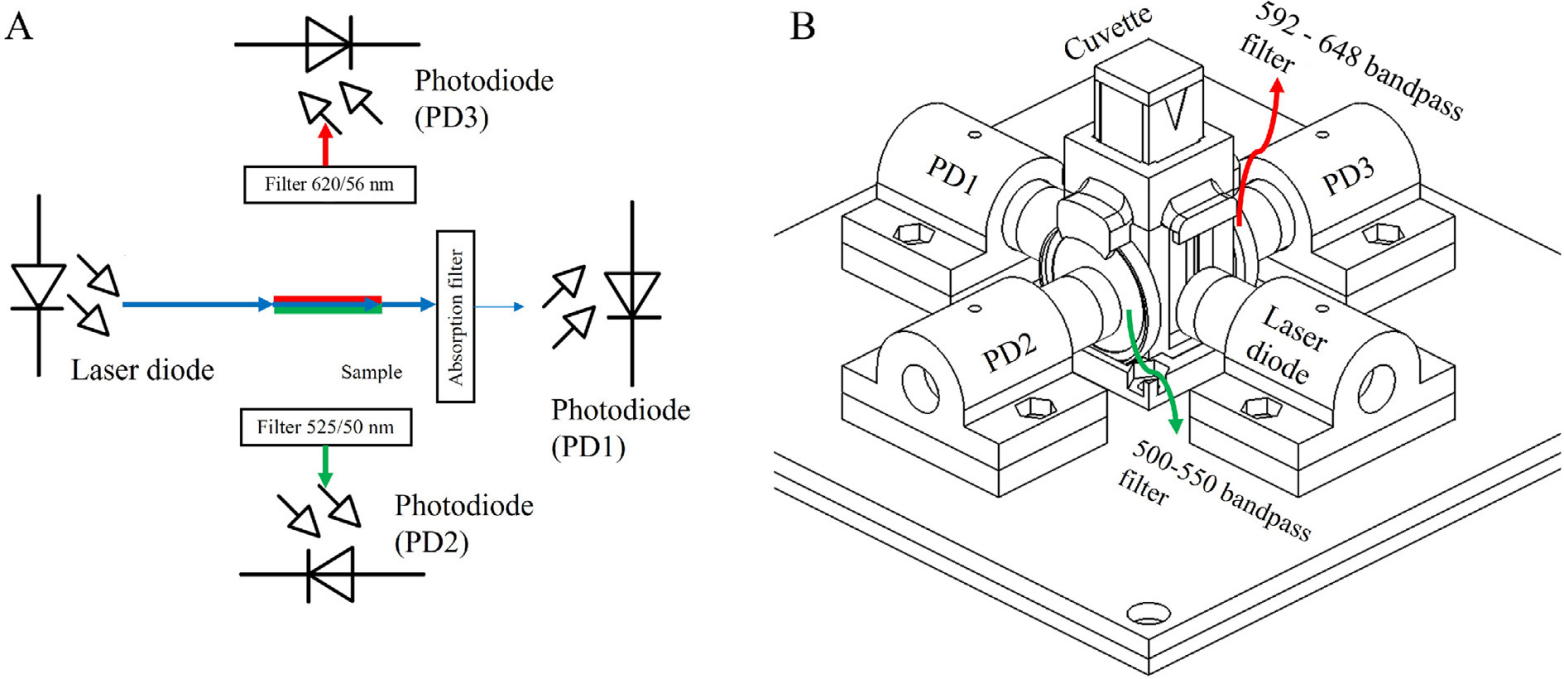
(A) The diagram and (B) the sketch of the fluorometer and absorption spectrometer setup inside the developed portable device.

In the current setup, a 450 nm laser diode (PLT5 450B, Osram Opto Semiconductor, USA) is focused by a plano-convex lens (4.87 mm lens diameter, 1.76 mm thickness, 7.15 mm focal length and 3.74 mm aperture stop diameter) into a cuvette containing the sample (Fig. 1). We chose a laser instead of light emitting diodes (LED) to minimize the cross-talk of the excitation light into the fluorescence emission channel and to avoid using excitation filters.

The semi-micro cuvette being used is disposable yet made from high quality polymethyl methacrylate (PMMA) (97000–590, VWR, U.K.) suitable for working with light having wavelengths between 300 nm and 900 nm. The internal dimensions are 10.0 mm × 4.0 mm (length × width) while the outer dimensions are 12.5 mm × 12.5 mm × 45.0 mm (length × width × height). Cheaper alternatives, such as microcentrifuge tubes were tested but the semi-micro cuvette offered a much better reproducibility with only a slight extra cost. Consequently, our portable device accepts a regular full-size (outer dimensions of 12.5 mm × 12.5 mm × 45.0 mm (length × width × height)) fluorescence cuvette as well.

There are three photodiodes (PS1.0–5-TO52S1, First Sensor, Germany) for light detection. Photodiode 1 (PD1) is for measuring light absorption (at 450 nm in the current setup) after passing through the sample’s cuvette. If needed, the light intensity can be reduced to the sensitivity range of the photodiode by a low-cost poly (methyl methacrylate) 525 nm cut-on absorption filter which is mounted in front of PD1*.* Photodiode 2 (PD2) and 3 (PD3) are for detecting fluorescence emission at 90 degrees relative to the direction of the excitation beam in dual wavelength ranges. As a demonstration, we place a 500–550 bandpass filter in front of PD2 to detect green fluorescence (e.g. from fluorescein) and a 592–648 bandpass filter in front of PD3 to detect orange-red fluorescence (e.g. from tetramethylrhodamine (TMR)). Each photodiode is mounted in a preassembled housing with another plano-convex lens (4.87 mm lens diameter, 1.76 mm thickness, 7.15 mm focal length and 3.74 mm aperture stop diameter) to maximize incident light intensity. Even though there are many cheaper photodiodes, such as the BPW34, available, the PS1.0–5-TO52S1 appears to be the best choice for constructing our device. When compared with the BPW34, the responsivity at 550 nm (close to the peak wavelength of fluorescein) from the PS1.0–5-TO52S1 is twice as much. In addition, the PS1.0–5-TO52S1 provided 0.2 nA dark current and 1.5 × 10^-14^ W/Hz^½^ noise equivalent power which are 10 and 2.66 times lower than that of the BPW34, respectively. Therefore, the BPW34 is still inferior to the PS1.0–5-TO52S1 in the case of fluorescence measurement applications, but it may be used in absorbance measurement applications.

### Signal conditioning

The dynamic range of detection can be easily adjusted by varying the laser intensity and the amplifying gain of photodiode. The laser intensity is proportional to the current fed from a low-cost laser driver power supply 100mW-3 W 12VDC. The current setting can be done in two ways. The first was by directly limiting the current via a variable resister. In this mode, the TTL (transistor-transistor logic) port was set to 5 V to enable the current feeding, and the intensity of the laser can be set at a desirable value (e.g., 100 mW in our case) by manually adjusting the resister*.* The second method uses PWM (pulse width modulation) generated from an Arduino-compatible board (Mega 2560 Pro, RobotDyn, China) at the TTL port for fine light intensity adjustment.

The photodiodes are operated in reverse bias by a laboratory-built circuit. The reverse bias (at −20 V) forces the photodiode to operate in the photocurrent mode, in which the output current is linearly proportional to the incident light intensity. Also, the reverse bias gives a better responsivity compared with the zero bias (photovoltaic mode). The photocurrent output is converted into voltage signal and amplified via an operational amplifier based transimpedance amplifier*.* Finally, the signals were adjusted to match the analogue to digital circuit (ADC) dynamic range for data acquisition. The conversion rate for the ADC is set at 1,600 times per second or approximately 0.625 ms accumulation time. This ADC conversion rate was chosen so that the accumulation time is well (approximately 25 times) shorter than the laser on time in each PWM cycle. The op-amp being used (LM318) is capable of amplifying the signal with frequency ranging from DC to 15 MHz (i.e., 15 MHz bandwidth). Therefore, the ADC and op-amp are compatible.

The key features of the device are as follow:

- Capable of dual detection of fluorescence and light absorption in one portable device.
- Contains no moving parts, inexpensive, customizable and easy to operate with minimal maintenance.
- Works with sample volume as small as 500 μl.

## Design files summary

### Bill of materials summary

#### Build instructions

The optical breadboard and device’s body frame was 3D printed using a fused deposition modeling (FDM) printer (Chiron, Anycubic, China). The circuit mainboard was engraved by a computer numerical control (CNC) router machine (3018 mini-CNC Grbl Control Systems, CRONOS MAKER, China).

## Additional items necessary for fabrication and assembly

- soldering iron and solder
- drill press
- screw drivers
- epoxy
- sand papers
- pliers
- double sided tape
- laser safety glasses
- power meter (e.g., PM100D, ThorLabs, USA)
- USB to serial UART interface (e.g., FT232RQ, Future Technology Devices International Ltd, China)
- computer

### Hardware fabrication

#### 3D printing

1. In the 3D printing slicer software, e.g., Ultimaker Cura, import selected Parts (1–17) (Table 1) from the STL files and orient them on the working space as shown in Fig. 2A-E.

**Table 1:** Design files summary.

**Design file name**	**File type**	**Open source license**	**Location of the file**
Part 1	STL file; FCstd file	CC	https://doi.org/10.17632/bdg2c7tsxm.1
Part 2	STL file; FCstd file	CC	
Part 3	STL file; FCstd file	CC	
Part 4	STL file; FCstd file	CC	
Part 5	STL file; FCstd file	CC	
Part 6	STL file; FCstd file	CC	
Part 7	STL file; FCstd file	CC	
Part 8	STL file; FCstd file	CC	
Part 9	STL file; FCstd file	CC	
Part 10	STL file; FCstd file	CC	
Part 11	STL file; FCstd file	CC	
Part 12	STL file; FCstd file	CC	
Part 13	STL file; FCstd file	CC	
Part 14	STL file; FCstd file	CC	
Part 15	STL file; FCstd file	CC	
Part 16	STL file; FCstd file	CC	
Part 17	STL file; FCstd file	CC	
Part 18	nc file	CC

**Fig. 2:**
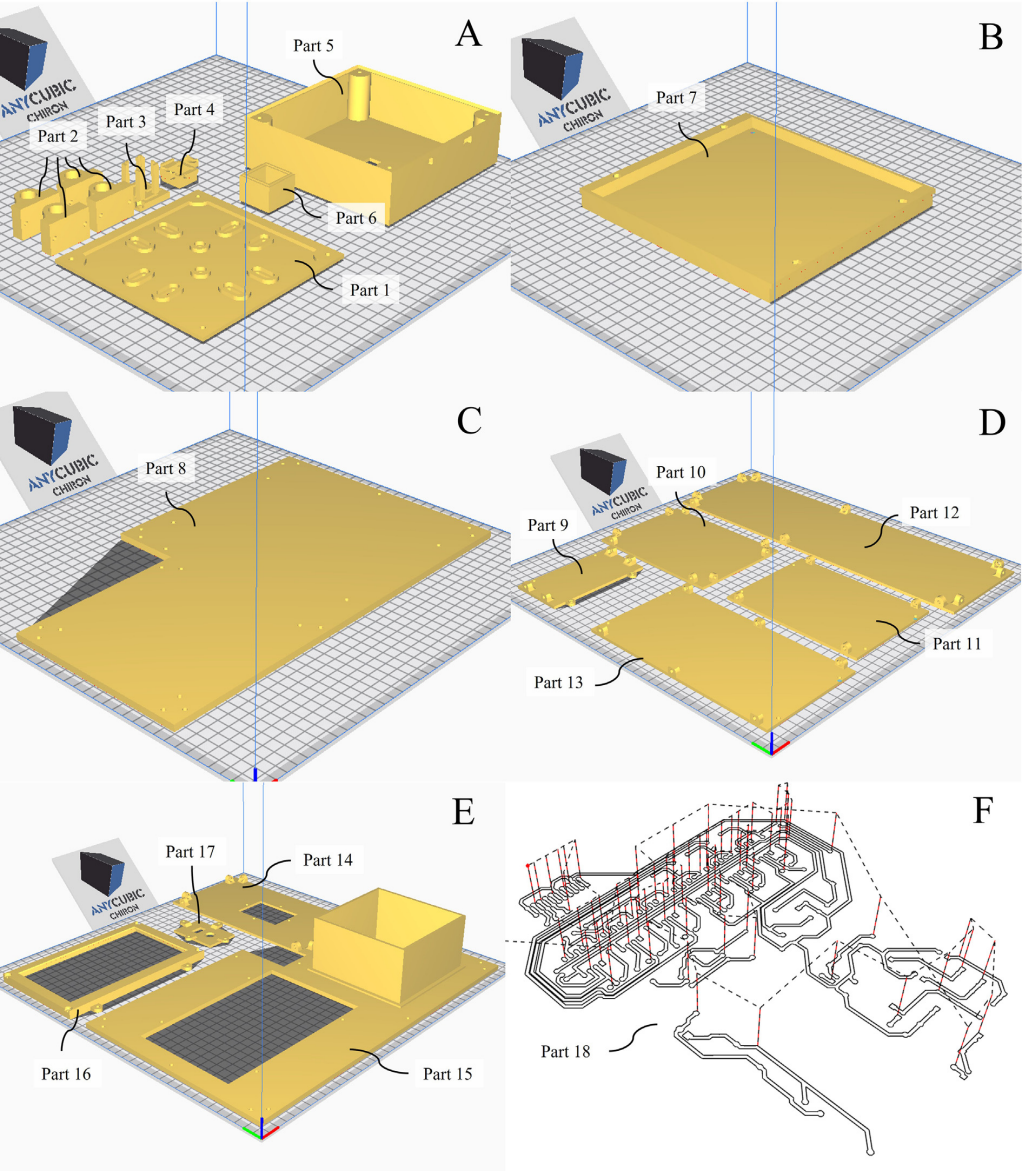
(A to E) Orientation of Part 1 to 17 on the building plate in the 3D printing slicer software. (F) Travelling paths of a V-shape cutting bit on the printed circuit board (PCB).
2. Set layer height to 0.2 mm and line width to 0.38 mm.
3. Set wall thickness to 1.6 mm and Infill density to 20% to ensure the durability of all parts.
4. Set support pattern to Gyroid for an easy removal.
5. Create g-code file, upload to the 3D printer, and print the parts.
6. If necessary, use desktop drilling machine to drill holes, particularly Part 9 to 13.

#### Engraving

1. Mount a copper clad laminate (CCL) plate onto the cutting table of the CNC router machine using a double-sided tape. Make sure that the dimensions of the CCL plate are at least 2 cm. bigger than the dimensions of the circuit pattern being engraved.
2. Mount a 30-degree V-shape cutting bit into the spindle.
3. Lower the spindle until the tip of the V-shape bit touches the CCL surface. The tip of the bit should be at the center of the CCL plate. Set zero value to all axes.
4. Upload the **Part 17_circuit.nc** file to a CNC compiler, such as Candle, and start engraving along the designed path (Fig. 2F).
5. Replace the V-shape cutting bit to a 3 mm end mill bit in order to cut out the engraved PCB.
6. Lower the spindle until the tip of the 3 mm endmill bit touches the CCL surface and set zero value to all axes.
7. Upload **Part 17_pcbcutting.nc** file to CNC compiler and start cutting.
8. Drill holes for through-hole mounting of electronic components using the drill press.

#### Mainboard assembly

**Fig. 3:**
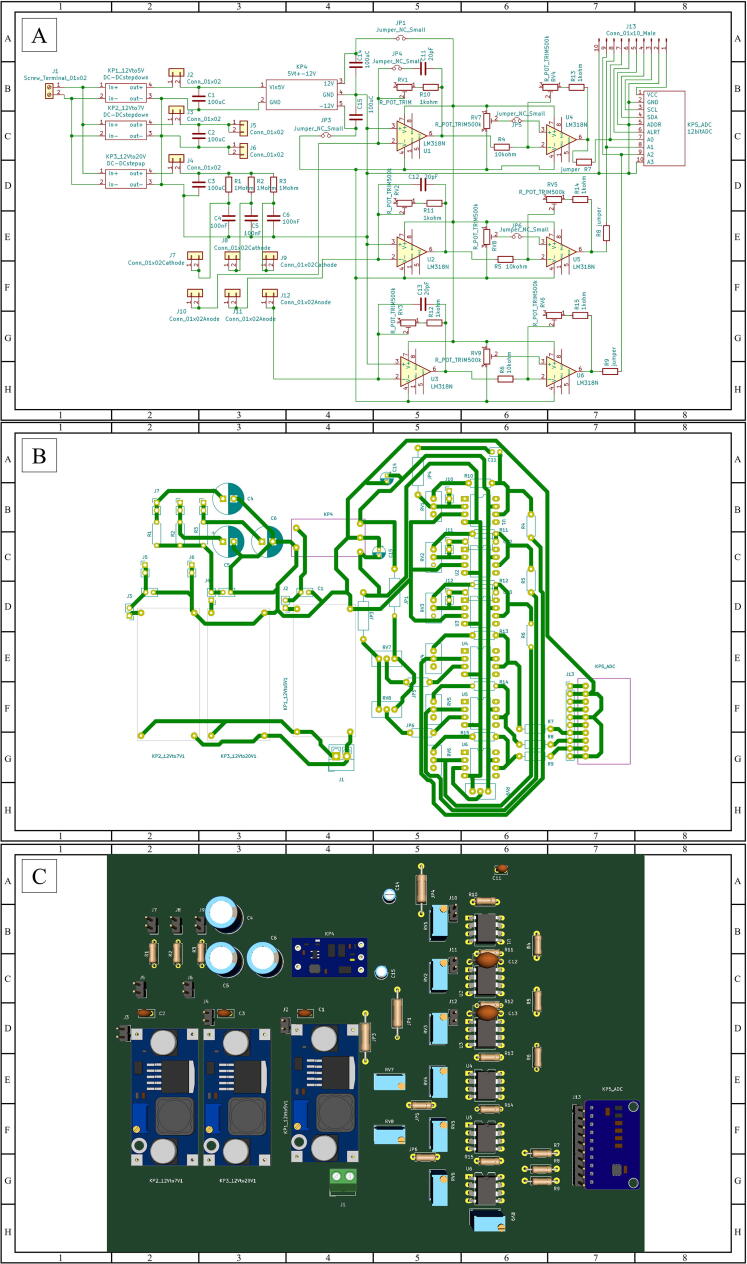
(A) Circuit schematic diagram, (B) circuit pattern on the printed circuit board, and (C) 3D image of the finished mainboard.
- Solder the through hole resistors and capacitors onto the engraved PCB. Note that the polarity of the electrolyte capacitors is important. Soldering time for a joint should not exceed 10 s. Trim the component leads if necessary.
- Solder the operational amplifiers (op-amp) onto the PCB. Make sure that the orientation the op-amps on the PCB is done correctly according to Fig. 3B and C. Pin 1 can be found next to the marker on the op-amp package (when viewed from the top). The recommended soldering temperature and time is 230  °C and 10 s, respectively.
- Install the DC-to-DC Step Up module (XL6090, at E3 in Fig. 3C), DC-to-DC Step Down module (LM2596, at E2 and E4 in Fig. 3C), Dual poles DC-DC Step Up module (Signal Power supply module, at B4 in Fig. 3C), and ADC module (ADS1015, at F7 in Fig. 3C) on the PCB.
- Split a 1x40 pin header in to 11 pieces of 1x2 pin header and 1 piece of 1x10 pin header. Install the 1x2 pin headers (3 pieces at B2, 2 pieces at C2, 1 piece each at D2, D3, D4, B5, C5 and D5 in Fig. 3C, respectively), the 1x10 pin header (at F7 in Fig. 3C) and the terminal connector J1 (at G4 in Fig. 3C) on the PCB.

1. Solder all electronic components on the engraved PCB. All parts are listed in Table 2 (Bill of materials summary) and their connections are shown in the circuit schematic diagram in Fig. 3A. The actual positions of each component on the PCB are as shown in Fig. 3B-C. Any 20 W-130 W soldering iron (e.g., No.981, Hakko Technologies Sdn Bhd, Japan) and lead-free or lead alloy solder (e.g., 0.7 mm RS756-8904, RS Components Ltd., U.K.), which are widely available, can be used. The steps should be as follows.

**Table 2:** Bill of materials summary.

**Designator**	**Component name**	**Quantity**	**Cost/unit (USD)**	**Total cost(USD)**	**Source of materials**	**Alternative component**	**Source of alternative materials**	**Material type**
Part 1–17		1	18.15/L	27.37	https://www.print3dd.com/product/flashforge-pla-1–75 mm/	N/A	N/A	Flashforge PLA 1.75 mm
Part 18	PCB	1	1.99/sheet	1.99	https://www.analogread.com/product/406/%E0%B9%81%E0%B8%9C%E0%B9%88%E0%B8%99%E0%B8%97%E0%B8%AD%E0%B8%87%E0%B9%81%E0%B8%94%E0%B8%87%E0%B8%AA%E0%B8%B3%E0%B8%AB%E0%B8%A3%E0%B8%B1%E0%B8%9A%E0%B8%81%E0%B8%B1%E0%B8%94%E0%B8%A5%E0%B8%B2%E0%B8%A2%E0%B8%A7%E0%B8%87%E0%B8%88%E0%B8%A3	N/A	N/A	Single Sided Copper Coated PCB
Part 19	20 × 10 × 1 mm^3^ absorption filter	1	36.74/sheet	0.03	A local store	N/A	N/A	Transparent orange acrylic sheet
Part 20	20 × 10 × 0.03 mm^3^ absorption filter	1	17.50/sheet	0.01	A local store	N/A	N/A	304 Stainless Steel Filter Mesh Screen
Part 21	20 × 10 × 1 mm^3^ absorption filter	1	36.74/sheet	0.03	A local store	N/A	N/A	Transparent acrylic sheet
Laser diode	Laser Diodes Blue Laser PLT5 450B450nm, 100mW	1	34.94	34.94	https://th.mouser.com/ProductDetail/OSRAM-Opto-Semiconductors/PLT5-450B?qs = P%252BGkiCIryJtXEw%252BMukmdsg%3D%3D	Laser Diodes Blue laser PL 450B	https://th.mouser.com/ProductDetail/OSRAM-Opto-Semiconductors/PL-450B?qs = Wn16VcyqZWqfO9UTIm230Q%3D%3D	Other
5mW red laser module	Lens and housing	4	1.99	7.96	https://www.analogread.com/product/836/5mw-red-laser-module	N/A	N/A	Other
Photodiode	Photodiodes 1 mm squared PIN detector Blue/Green PS*1.0*–*5* TO*52S1*	3	31.97	95.92	https://th.mouser.com/ProductDetail/First-Sensor/PS10-6B-TO52S13?qs = hIohqi1S7IpnxK5X5xc9dg%3D%3D	PC1-6-TO52S3	https://lv.pcbekey.com/products/1_252669/pc1-6-to52s3	Other
Bandpass filter	525 nm CWL, 25 mm Dia. Hard Coated OD 4.0 50 nm Bandpass Filter	1	222.30	222.30	https://www.edmundoptics.com/p/525 nm-cwl-25 mm-dia-hard-coated-od-4–50 nm-bandpass-filter/29363/	525/50 nm BrightLine® single-band bandpass filter	https://www.semrock.com/FilterDetails.aspx?id = FF03-525/50–25	Optical Glass
Bandpass filter	620 nm CWL, 25 mm Dia, 56 nm Bandwidth, OD 6 Fluorescence Filter	1	282.93	282.93	https://www.edmundoptics.com/p/620 nm-cwl-25 mm-dia-56 nm-bandwidth-od-6-fluorescence-filter/3364/	Bandpass Filter, Fluorescence, 25.0 mm, 620 nm Center, 60 nm Bandwidth	https://www.newport.com/p/HPM620-60	Fused Silica Windows
Laser driver	Power Supply 100mW-3 W 12VDC for 405–450 nm with TTL	1	5.82	5.82	https://www.zonemaker.com/product/1755/laser-driver-power-supply-100mw-3w-12vdc-for-405–450-nm-with-ttl	N/A	N/A	Other
Arduino micro-controller	Mega 2560 Pro (Embed)CH340G	1	11.02	11.02	https://www.analogread.com/product/1805/mega-2560-pro-embedch340g-arduino-compatible-board	Arduino mega 2560	https://www.analogread.com/product/3/mega-2560-r3-%E0%B9%81%E0%B8%96%E0%B8%A1%E0%B8%AA%E0%B8%B2%E0%B8%A2-usb-arduino-compatible-board-2	Other
Touch screen LCD	Nextion NX8048T070 7 in.	1	87.27	87.27	https://www.analogread.com/product/2171/nextion-nx8048t070-7-inch	Nextion NX8048K070 7 in.	https://www.thaieasyelec.com/product/449/nextion-enhanced-generic-7-inch-hmi-touch-display-nx8048k070?gclid = EAIaIQobChMI9LCrg5Xh9AIVhyQrCh2KwAUDEAQYASABEgIohPD_BwE	Other
ADC (Analogue to digital convertor) (KP5_ADC)	ADS1015 12-Bit ADC − 4 Channel with Programmable Gain Amplifier	1	3.67	3.67	https://www.analogread.com/product/803/ads1015-12-bit-adc-4-channel-with-programmable-gain-amplifier-%E0%B9%82%E0%B8%A1%E0%B8%94%E0%B8%B9%E0%B8%A5%E0%B8%AD%E0%B9%88%E0%B8%B2%E0%B8%99%E0%B8%84%E0%B9%88%E0%B8%B2-analog-%E0%B9%81%E0%B8%9A%E0%B8%9A-4-%E0%B8%8A%E0%B9%88%E0%B8%AD%E0%B8%87-%E0%B8%84%E0%B8%A7%E0%B8%B2%E0%B8%A1%E0%B8%A5%E0%B8%B0%E0%B9%80	N/A	N/A	Other
DC-DC Step Up (KP3_12Vto20V)	DC-to-DC Step Up XL6009 Module	1	1.80	1.80	https://www.analogread.com/product/608/dc-to-dc-step-up-xl6009-module-4a-%E0%B9%82%E0%B8%A1%E0%B8%94%E0%B8%B9%E0%B8%A5%E0%B9%81%E0%B8%9B%E0%B8%A5%E0%B8%87%E0%B9%84%E0%B8%9F%E0%B8%82%E0%B8%B6%E0%B9%89%E0%B8%99-%E0%B8%AA%E0%B8%95%E0%B9%87%E0%B8%AD%E0%B8%81%E0%B9%84%E0%B8%97%E0%B8%A2%E0%B8%AA%E0%B9%88%E0%B8%87%E0%B9%84%E0%B8%A7	N/A	N/A	Other
Dual poles DC-DC Step Up (KP4)	Signal Power supply module 2.8 V ∼ 5.5 V to positive and negative + -12 V	1	1.99	1.99	https://www.allnewstep.com/product/1419/signal-power-supply-module-2-8v-5-5v-to-positive-and-negative-12v-step-up-%E0%B9%81%E0%B8%9B%E0%B8%A5%E0%B8%87%E0%B9%84%E0%B8%9F-2–8-5-5v-%E0%B9%80%E0%B8%9B%E0%B9%87%E0%B8%99-%E0%B9%84%E0%B8%9F%E0%B8%9A%E0%B8%A7%E0%B8%81	N/A	N/A	Other
DC-DC Step Down (KP2_12Vto7V and KP1_12Vto5V)	DC-to-DC Step Down LM2596 Module	2	0.92	1.84	https://www.analogread.com/product/254/dc-to-dc-step-down-lm2596-module-3a-%E0%B9%82%E0%B8%A1%E0%B8%94%E0%B8%B9%E0%B8%A5%E0%B8%A5%E0%B8%94%E0%B9%81%E0%B8%A3%E0%B8%87%E0%B8%94%E0%B8%B1%E0%B8%99%E0%B9%84%E0%B8%9F%E0%B8%9F%E0%B9%89%E0%B8%B2-%E0%B8%88%E0%B9%88%E0%B8%B2%E0%B8%A2%E0%B8%81%E0%B8%A3%E0%B8%B0%E0%B9%81%E0%B8%AA%E0%B9%84%E0%B8%94%E0%B9%89%E0%B8%AA%E0%B8%B9%E0%B8%87%E0%B8%AA%E0%B8%B8%E0%B8%94-3a-%E0%B8%AA%E0%B8%95%E0%B9%87%E0%B8%AD%E0%B8%81%E0%B9%84%E0%B8%97%E0%B8%A2%E0%B8%AA%E0%B9%88%E0%B8%87%E0%B9%84%E0%B8%A7	N/A	N/A	Other
Operational amplifier (U1-U6)	LM318	8	0.92	7.35	https://www.es.co.th/detail.asp?Prod = 007801750	NE5534	https://th.rs-online.com/web/p/op-amps/0810217p?cm_mmc = TH-PLA-DS3A-_-google-_-PLA_TH_EN_Semiconductors_Whoop-_-(TH:Whoop!) + Op + Amps-_-810217P&matchtype=&aud-821594433763:pla-1297969549405&gclid = EAIaIQobChMI9Y65odvn9AIVRjErCh34dghGEAQYASABEgIyRPD_BwE&gclsrc = aw.ds	Other
Cable	24AWG Cable	1 roll	3.67	3.67	https://www.analogread.com/product/4139/%E0%B8%AA%E0%B8%B2%E0%B8%A2%E0%B9%84%E0%B8%9F%E0%B8%AA%E0%B8%B5%E0%B9%81%E0%B8%94%E0%B8%87-24awg-%E0%B8%A1%E0%B9%89%E0%B8%A7%E0%B8%99	N/A	N/A	Other
Pin header (J1-J13)	Pin Header Dip Straight Single Row 1X40PIN	2	0.15	0.31	https://www.analogread.com/product/285/pin-header-dip-straight-single-row-1x40pin-%E0%B8%81%E0%B9%89%E0%B8%B2%E0%B8%87%E0%B8%9B%E0%B8%A5%E0%B8%B2	N/A	N/A	Other
Plug	DuPont plug terminal core	6	0.21	1.29	https://www.analogread.com/product/309/dupont-plug-terminal-core-%E0%B8%88%E0%B8%B3%E0%B8%99%E0%B8%A7%E0%B8%99–10-%E0%B8%95%E0%B8%B1%E0%B8%A7	N/A	N/A	Other
Heat shrink tube	3 mm diameter Heat shrink tube	1	0.15	0.15	A local store	N/A	N/A	Other
Switch	Switch ON – Off 8.5*13.5 mm	1	0.15	0.15	https://www.analogread.com/product/899/switch-on–off-%E0%B8%82%E0%B8%99%E0%B8%B2%E0%B8%94%E0%B9%80%E0%B8%A5%E0%B9%87%E0%B8%81–8-513–5 mm	N/A	N/A	Other
Power socket	DC power socket	1	0.15	0.15	https://www.analogread.com/product/613/dc-power-socket-5–5-2–1-mm-%E0%B8%95%E0%B8%B1%E0%B8%A7%E0%B8%96%E0%B8%B1%E0%B8%87%E0%B8%A1%E0%B8%B5%E0%B9%80%E0%B8%81%E0%B8%A5%E0%B8%B5%E0%B8%A2%E0%B8%A7	N/A	N/A	Other
Variable resistance (RV1-RV9 labels in Fig. 3)	3296 W Trimpot Variable Resistor (500kΩ)	10	0.31	3.06	https://www.analogread.com/product/1750/%E0%B8%95%E0%B8%B1%E0%B8%A7%E0%B8%95%E0%B9%89%E0%B8%B2%E0%B8%99%E0%B8%97%E0%B8%B2%E0%B8%99%E0%B8%9B%E0%B8%A3%E0%B8%B1%E0%B8%9A%E0%B8%84%E0%B9%88%E0%B8%B2%E0%B9%84%E0%B8%94%E0%B9%89–500 k-%E0%B8%AB%E0%B8%A1%E0%B8%B8%E0%B8%99–25-%E0%B8%A3%E0%B8%AD%E0%B8%9A	N/A	N/A	Other
Electrolyte capacitor (C1-C3 and C14-C15 labels, Fig. 3)	Electrolytic Capacitors – 100uF 50 V	5	0.46	2.30	https://www.analogread.com/product/2710/electrolytic-capacitors-100uf-50v	N/A	N/A	Other
Ceramic capacitor (C4-C6)	Ceramic Capacitor Pack	1	1.52	1.52	https://www.analogread.com/product/336/ceramic-capacitor-pack	N/A	N/A	Other
Resistor (R1-R15)	Resistor Pack	1	1.84	1.84	https://www.analogread.com/product/328/resistor-pack-%E0%B8%AA%E0%B8%95%E0%B9%87%E0%B8%AD%E0%B8%81%E0%B9%84%E0%B8%97%E0%B8%A2%E0%B8%AA%E0%B9%88%E0%B8%87%E0%B9%84%E0%B8%A7	N/A	N/A	Other
Pin Header(J2-J13)	Pin Header Dip Straight Single Row 1X40PIN	1 row	0.15	0.15	https://www.analogread.com/product/285/pin-header-dip-straight-single-row-1x40pin-%E0%B8%81%E0%B9%89%E0%B8%B2%E0%B8%87%E0%B8%9B%E0%B8%A5%E0%B8%B2	N/A	N/A	Other
Jumper cap	Jumper cap 2 Pins Female Pitch 2.54 mm	1 pack	0.15	0.15	https://www.analogread.com/product/1069/jumper-cap-2-pins-female-pitch-2–54 mm-black	N/A	N/A	Other
Terminal Connector(J1)	2P Terminal Connector (Green) 3.5 mm	1	0.1	0.1	https://www.analogread.com/product/3799/2p-terminal-connector-green3-5 mm	N/A	N/A	Other
Bolt & Nut	Bolt M3*15 mm	4	0.55	2.20	https://www.analogread.com/product/108/bolt-m315-mm-frearson-%E0%B8%AB%E0%B8%B1%E0%B8%A7%E0%B8%AA%E0%B8%B5%E0%B9%88%E0%B9%81%E0%B8%89%E0%B8%81	N/A	N/A	Other

**Caution:** If using a lead alloy solder, make sure that the soldering is performed with chemical resistance gloves (e.g., PVC gloves) on in an area with good air ventilation. Avoid inhaling solder fume and wash hands thoroughly with soap and water after finish soldering.

2. Connect a 12 V 5A DC power to the 2P Terminal connector J1 (at G4 in Fig. 3C). The power line connects to the right terminal and the neutral line connects to the left terminal, respectively.
3. Connect a multimeter probe to the output terminal of the 12 V to 5 V DC-DC Step Down module (at E4 in Fig. 3C) and adjust the voltage output via the variable resistor on the DC-DC Step Down module until the output voltage is 5 V.
4. Do the same to the other DC-DC Step Down module (at E2 in Fig. 3C) and the DC-to-DC Step Up module (at E3 in Fig. 3C) to set the output voltages to 7 V and 20 V, respectively.
5. Cap the connector J2 (at D3 in Fig. 3C), J3 (at D2 in Fig. 3C) and J4 (at D3 in Fig. 3C) to connect the power source to all components on the PCB.
6. Connect the cathode of PD1, PD2 and PD3 to a pin on the connector J7, J8 and J9 (at B2 in Fig. 3C), respectively. Then connect the anode of PD1, PD2 and PD3 to a pin on the connector J10, J11 and J12 (at B5 to D5 in Fig. 3C), respectively.
7. Connect a pin of the connector J5 and J6 (at C2 in Fig. 3C) to Vin and GND pins of the Arduino Mega. This powers the microcontroller.
8. Connect the I2C port of the ADS1105 to the I2C port of the Arduino Mega.

### Hardware

#### Optical setup

**Fig. 4:**
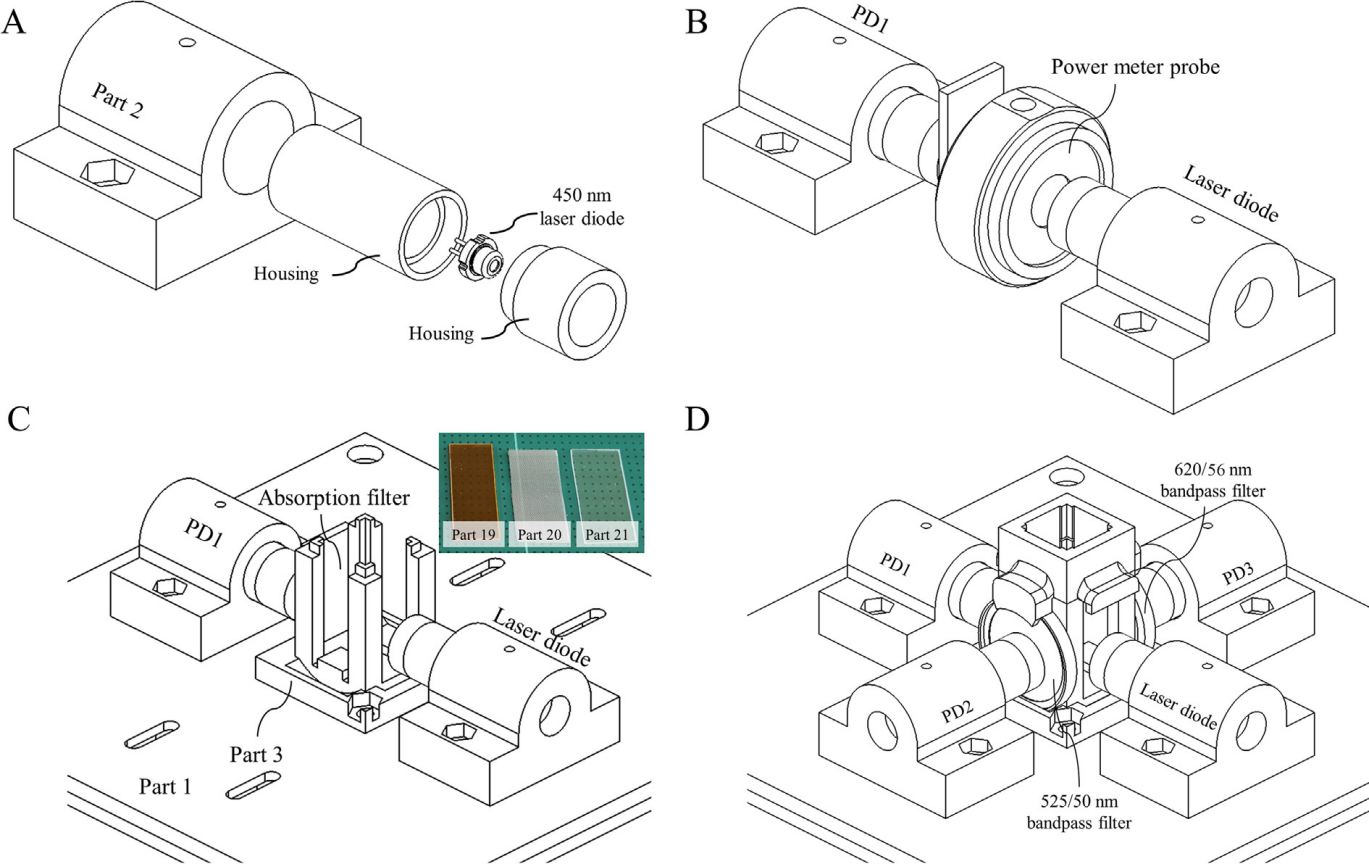
Sketches of (A) the laser diode installation, (B) the laser diode calibration and alignment, (C) the absorption measurement setup and (D) the fluorescence measurement setup.

In order to minimize the cost and assembly effort, we used pre-assembled housings and lens from commercial 5 mW red laser modules (e.g., SYD1230, Howard Batchen, UK) which are relatively inexpensive and widely available as the housing and focusing lens for our 450 nm laser diode and photodiodes. For the laser, we carefully replaced the red laser diode with our 450 nm laser diode. In the case of photodiodes, the laser housing has to be enlarged to 5 mm to match the photodiode’s diameter. After that, the photodiode and laser diode housings were installed in the Part 2 before being mounted on the printed optical breadboard as shown in Fig. 4A.

Next, the optical setup for the absorption measurement was performed as follows:

- Cover photodiode 1 (PD1, Fig. 1) to prevent unnecessary light exposure during the setup.
- Connect the laser diode and PD1 to their drivers.
- Re-install the focusing lens to the laser diode housing and adjust the lens position until a parallel laser beam is obtained.
- Set the TTL signal on the laser drive to 5 V before adjusting the variable resistor until the laser power is 100 mW (on the power meter equipped with a neutral density filter) (Laser safety glasses for eye protection are required)
- Uncover PD1 so that the laser is incident on the photodiode. Adjust the position and tilt angles of the photodiode until the output signal is maximum. Secure PD1.
- Re-install the focusing lens to maximize the signal.
- Install the sample holder on the optical breadboard and place a stack of absorption filters (Part 19, Part 20, and Part 21) in the slot (Part 3) in front of the PD1.

Next, the optical setup for the fluorescence measurement was performed as follows:

- Connect the laser diode and photodiode 2 and 3 (PD2 and PD3) to their drivers.
- Re-install the focusing lens to PD2 and PD3 housings. Place the 525/50 nm bandpass filter and 620/60 nm bandpass filter in the slots in front of PD2 and PD3, respectively (Fig. 4C)
- Install and glue Part 4 to the sample holder.
- Fill a standard fluorescein solution (1.000 µM fluorescein in 1x TE (10 mM Tris and 1 mM EDTA) buffer pH 8.0) in a microcuvette and put the cuvette in the sample holder.
- Adjust the positions and angles of PD2 and PD3 until the output signals reached maximum values.
- Connect the TTL signal pin of the laser driver to the D5 pin of the Arduino board.

**Caution:** Proper and certified laser safety glasses must be used at all time when working with laser. In this work, we used Thorlabs’ laser safety glasses (LG3, Thorlabs Inc., U.S.A.) that effectively block the 450 nm blue light (OD_450_ > 7.0) from the laser diode while allowing some light (with wavelength longer than 550 nm) emitted from the sample to be observed. In addition, during assembling and testing the device, avoid wearing or using personal accessories with glossy surfaces, such as a wristwatch or a smartphone.

#### Electrical condition

The output signal from each photodiode has to match the dynamic range of the ADC module, therefore the signal conditioning was required. Photocurrents generated by the photodiodes were converted to voltage and amplified via an op-amp based transimpedance amplifier. The amplifying gain was adjusted from a negatively feedbacked resistor to obtain measurable signal. The voltage signal was passed through an inversing amplifier with an offset to match the dynamic range of the ADC module and stored in the microcontroller. Note that even though the photodiodes (PS1.0–5-TO52S1) and op-amp (LM318) are designed for high-speed measurements, our device operates only in a stationary mode. The rationale behind choosing the photodiodes is based purely on their high responsivity to fluorescence from our model fluorophore (i.e., fluorescein), low dark current, wide availability, and low price. The op-amp was chosen based on the compatibility with the selected photodiodes.

#### System calibration

For fluorescence measurement

Prepare a “Fluorescent sample” by filling a semi-microcuvette with 500 µl of 1.000 µM fluorescein in 10 mM Tris and 1 mM EDTA (1 × TE) buffer solution pH 8.0. A “Blank sample” is prepared in the same way but without fluorescein present in the solution. Next, the calibration of fluorescence detection is performed as follows:

- With the device switched off, set the multimeter to measure resistance and connect the positive and negative probes to the inverting input (pin 2) and output (pin 6) of the op-amp U1 (at B6 in Fig. 3C) and adjust the variable resister RV1 (at B5 in Fig. 3C) until the resistance value shown on the multimeter is 500 kΩ.
- Repeat the procedure with the op-amp U2 (at C6 in Fig. 3C) and the variable resistor RV2 (at C5 in Fig. 3C) until the resistance value shown on the multimeter is 500 kΩ.
- Repeat the procedure with the op-amp U3 (at D6 in Fig. 3C) and the variable resistor RV3 (at D5 in Fig. 3C) until the resistance value shown on the multimeter is 200 kΩ.
- Switch the device on.
- Set the D5 pin of the Arduino board to 5 V to turn the laser on.
- Set the multimeter to measure DC voltage and connect the positive and negative probes to pin 7 and pin 2 (counting from the top) of the connector J13 (at F7 in Fig. 3C).
- Place the cuvette containing the “Blank sample” in the sample holder.
- Adjust the variable resistor RV7 (at E5 in Fig. 3C) until the voltage displayed on the multimeter is 0.00 V. This sets the zero value in Channel 1 (with PD2 as the detector) of the fluorescence measurements.
- Connect the positive and negative probes of the multimeter to pin 8 and pin 2 (counting from the top) of the connector J13 (at F7 in Fig. 3C).
- Replace the cuvette containing the “Blank sample” with the cuvette containing the “Fluorescent sample” in the sample holder.
- Adjust the variable resistor RV4 (at E5 in Fig. 3C) until the voltage displayed on the multimeter is 5.00 V. This sets the maximum value in Channel 1 (with PD2 as the detector) of the fluorescence measurements
- To calibrate Channel 2 (with PD3 as the detector) of the fluorescence measurements, first record the resistance values of the variable resistor RV4 and RV7 that have been set during Channel 1 calibration. Then, adjust the variable resistor RV5 (at F5 in Fig. 3C) until its resistance matches with that of RV4 and adjust the variable resistor RV8 (at F5 in Fig. 3C) until its resistance matches with that of RV7.

## For absorbance measurement

- With the device switched off, set the multimeter to measure resistance and connect the positive and negative probes to the inverting input (pin 2) and output (pin 6) of the op-amp U3 (at D6 in Fig. 3C) and adjust the variable resister RV3 (at D5 in Fig. 3C) until the resistance value shown on the multimeter is 200 kΩ.
- Switch the device on.
- Set the D5 pin of the Arduino board to 5 V to turn the laser on.
- Set the multimeter to measure DC voltage and connect the positive and negative probes to pin 9 and pin 2 of the connector J13 (at F7 in Fig. 3C). Read the voltage displayed on the multimeter; this is called V_air_. Place the cuvette containing the “Blank sample” in the sample holder. Read the voltage displayed on the multimeter; this is called V_blank._ If V_blank_ < V_air_, go to the next step. If not, keep adding an absorption filter in the mount in front of PD1 and repeat the process until V_blank_ < V_air_.
- Set D5 pin to 0 V to turn the laser off.
- Adjust the variable resistor RV9 (at H6 in Fig. 3C) until the voltage displayed on the multimeter is 0.00 V. This sets the zero value of the measured light intensity impinging on the photodiode that is later used to calculate absorbance measurements.
- Set the D5 pin of the Arduino board to 5 V to turn the laser on again.
- With the cuvette containing the “Blank sample” still in the sample holder, adjust the variable resistor RV6 (at G5 in Fig. 3C) until the voltage displayed on the multimeter is 5.00 V. This sets the maximum value of the measured light intensity impinging on the photodiode that is later used to calculate absorbance measurements.

### Signal conversion

The conversion from fluorescence intensity impinging on the photodiode to the final voltage output followed by the digitization by the ADC may be explained as follow:

**Step 1**: The conversion from the impinging fluorescence intensity to the photocurrent:

$$I_{\mathrm{photo}}=\gamma \cdot A\cdot F_{520}+I_{D}=2.7\times 10^{-7}\cdot F_{520}+2\times 10^{-10}$$

Where $I_{\mathrm{photo}}$ is the photocurrent (A)

$F_{520}$ is the fluorescence intensity measured at 520 nm, where fluorescein usually emits most brightly (W/m^2^)

γ is the responsivity of the photodiode at 520 nm (approximately 0.27 A/W, according to the data sheet from the manufacturer)

A is the active area of the photodiode (1 mm^2^)

$I_{D}$ is the dark current (0.2 nA, according to the data sheet from the manufacturer)

**Step 2**: The conversion from the photocurrent to a voltage by the transimpedance amplifier

$$V_{\mathrm{photo}}=-R\cdot I_{\mathrm{photo}}$$

Where $V_{\mathrm{photo}}$ is the voltage converted from the photocurrent (V)

R is the transimpedance gain

**Step 3**: The final amplification

$$V_{\mathrm{out}}=-G\cdot V_{\mathrm{photo}}-V_{\mathrm{offset}}$$

Where $V_{\mathrm{out}}$ is the output voltage before digitization (V)

$V_{\mathrm{offset}}$ is the offset voltage set during the calibration process (V)

G is the gain of the inverting amplifier

**Step 4**: The digitization is done by a 12-bit ADC. One level of the digital signal equals to 0.125 mV of $V_{\mathrm{out}}$.

### Maintenance

The developed device is designed to be simple to operate and easy to maintain. It is recommended that when not in use the lid of the sample holder compartment should be kept closed to prevent dust accumulation. If needed, a simple rubber dust blower can be used to remove dust particles from the sample compartment. In case of normal spillages, first switch the device off, remove the power cord from the outlet and then remove the spilled liquid with a piece of lint free cloth (or lint free tissue paper) followed by rubbing the spilled area with another piece of lint free cloth damped with clean water and let dry. In the case of persistent stain, rubbing the surface with a piece of lint free cloth damped with mild detergent or ethanol may be used before rubbing with another piece of lint free cloth damped with clean water and let dry. Avoid excessive washing.

#### Software

**Fig. 5:**
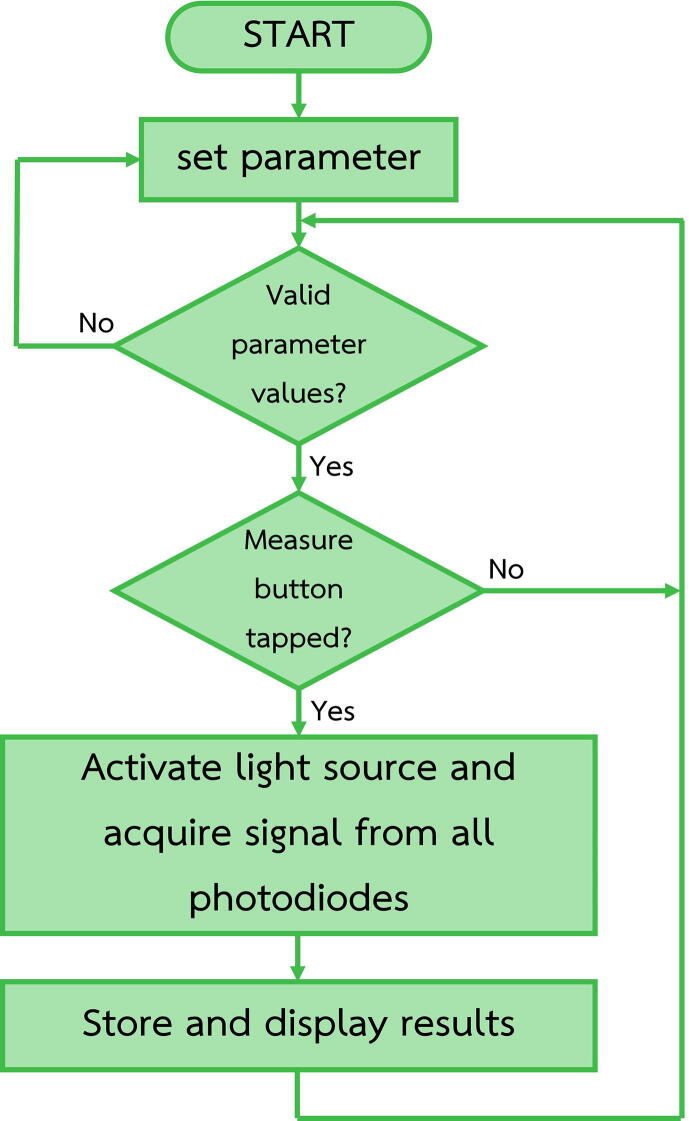
Flowchart describing the operation of the software running the device.

**Fig. 6:**
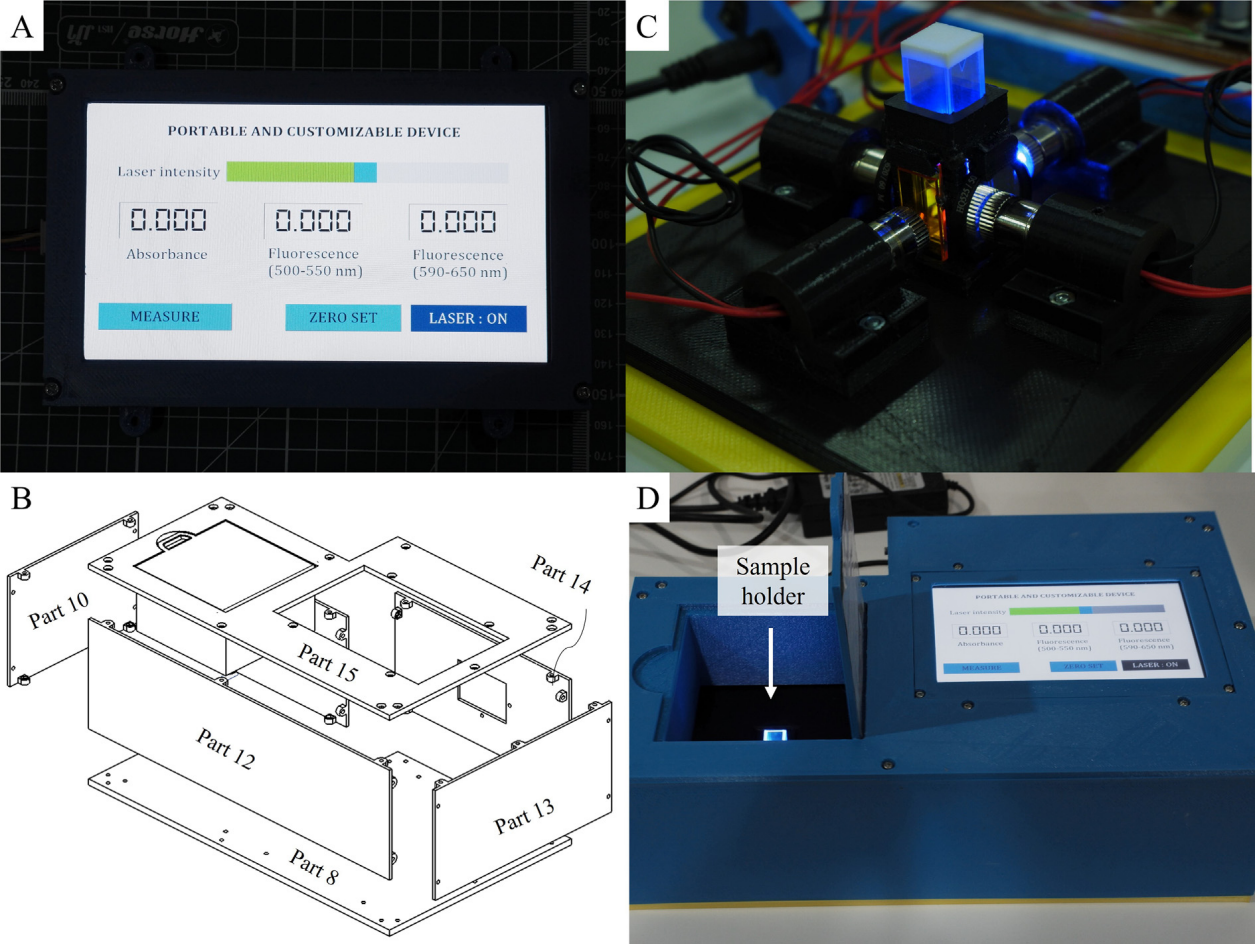
(A) photograph showing the touch screen on the device where the user set measurement parameters, start measurement and see the results. (B) sketch showing the assembly of Part 8 to 14. (C) photograph showing the assembly of the light source, the detectors and the sample holder. (D) photograph showing the complete device.

The software of the device works according to the flowchart shown in Fig. 5 and was developed in Arduino IDE (integrated development environment) (Arduino, Italy) and Nextion Editor (Nextion, China. The installation process is as follows.

a. Download and extract “Dual_Portable_Fluorescence_and_Absorption_Device.rar”. The file is located in the “Software” folder at https://doi.org/10.17632/bdg2c7tsxm.1
b. Download and install Arduino IDE (https://www.arduino.cc/en/software) and Nextion Editor (https://nextion.tech/nextion-editor/).
c. Add libraries for ADC and SD Cards module to Arduino IDE by following these steps.
  - Open Arduino IDE.
  - Press “Ctrl + Shift + I” to open the library manager.
  - In the topic search box, type “ADS1X15” for ADC modules.
  - Install the “Adafruit ADS1X15” library.
d. Open “Dual_Portable_Fluorescence_and_Absorption_Device.ino” in Arduino IDE.
e. Connect the Arduino Mega 2560 Pro to a computer using a micro-USB to USB cable.
f. Set the uploading environment in Arduino IDE as follows.
  Board: “Arduino Mega or Mega 2560”
  Processor: “ATmega2560 (Mega 2560)”
  Port: An appropriate serial port (can be checked at Device Manager from control panel of Windows operation system by checking at Ports (COM & LPT).
  For example, the port for our Arduino connection is shown as “USB-SERIAL CH340”.
g. Click ‘‘Upload” and wait until the ‘‘upload completed” message is shown.
h. Open file “Dual_Portable_Fluorescence_and_Absorption_Device_GUI.HMI” in Nextion Editor.
i. In the command panel, click “Device ID”, set the device to “basic” series and choose the model “NX8048T07_011”.
j. Connect the USB to serial UART module (e.g., FT232RL) to the UART port of the Nextion HMI display.
k. Click “Upload”.
l. Disconnect the UART wires from the USB to serial UART module and then make UART connection between the Arduino Mega 2560 pro and the Nextion HMI display.
m. After finishing the software installation, assemble all parts as shown in Fig. 6.
  1. Operation instructions

**Fig. 7:**
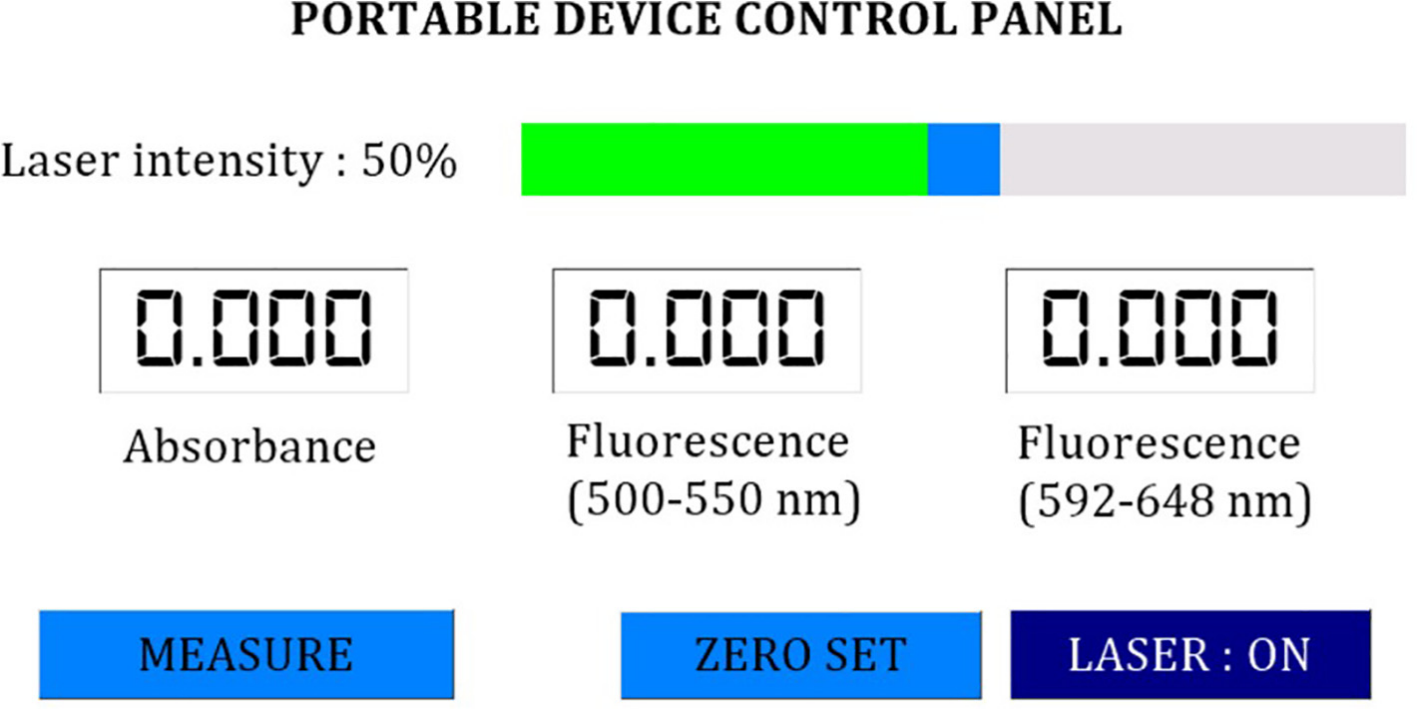
The control panel displayed on the screen of the device.
c. Open the sample holder cover, place the microcuvette inside and close the cover.
d. Press the “Measure” button to obtain absorbance at 450 nm or fluorescence emission in the wavelength range from 500 to 550 nm and 592 to 648 nm. The results are shown in the text boxes. (Fig. 6).

The device was designed to be simple and easy to use. Both absorbance and fluorescence measurements can be done using the same procedure as follows.

a. Fill at least 500 μl of sample solution in the microcuvette.
b. Set the desired intensity by input a value from 1 to 100 (mW of laser power) in the intensity slide bar (Fig. 7).

## Validation and characterization

The developed device was validated by comparing to results with that obtained from a UV–Vis spectrophotometer (U-2900, Hitachi, Japan) for absorbance measurement and a fluorescence spectrometer (F-2700, Hitachi, Japan) for fluorescence measurement using the same sets of samples.

### Absorbance measurement

**Fig. 8:**
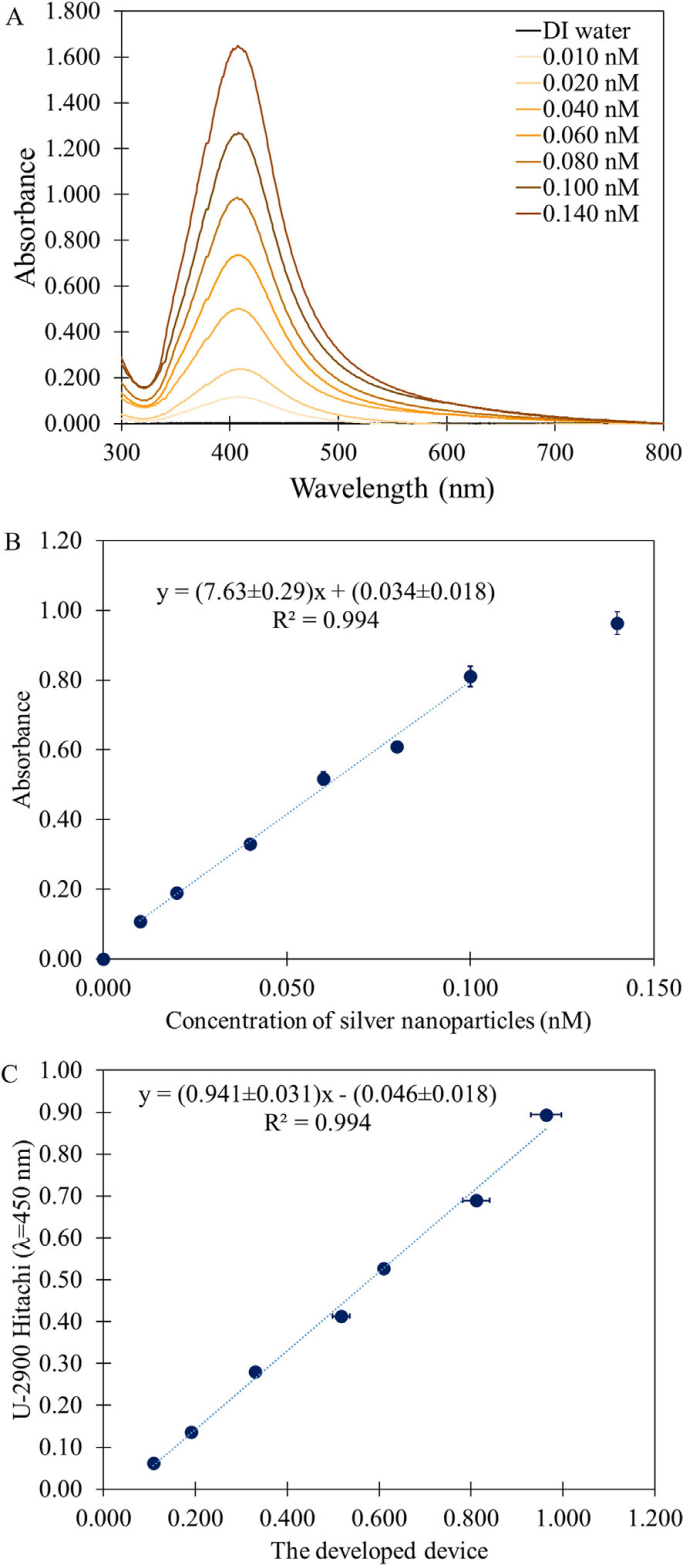
(A) Absorption spectra of silver nanoparticle solutions measured by a UV–Vis spectrophotometer (U-2900, Hitachi, Japan), (B) the absorbance from our portable fluorescence and absorption device and (C) a comparison between the absorbance at 450 nm from the commercial spectrophotometer and our portable device.

We chose solutions of silver nanoparticle (average size around 28 nm) as a model sample because nanoparticle-based optical sensors are very popular in which the concentration of targeted analytes were often quantified by changes in the absorbance of the solution [5], [6]*.* The solutions appear yellow indicating that they have strong absorption toward blue light. We varied the nanoparticle concentrations from 0.000 to 0.140 nM and the results from both the commercial UV–Vis spectrophotometer (Fig. 8A) and that from our device (Fig. 8B) increase with increasing nanoparticle concentration, as expected from the Beer-Lambert Law*.* Moreover, there is a good correlation between the two sets of results (Fig. 8C).

### Fluorescence measurement

**Fig. 9:**
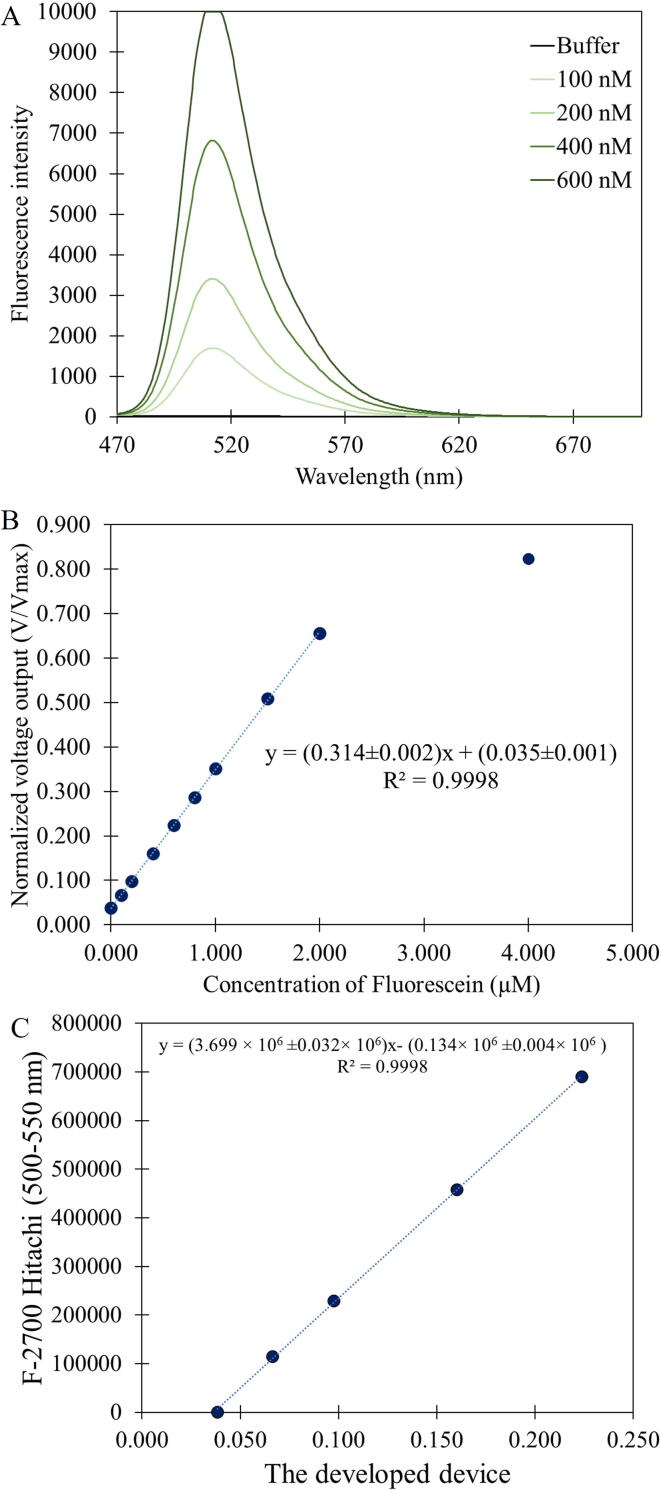
(A) Fluorescence emission spectra of fluorescein measured by a commercial fluorometer (F-2700, Hitachi, Japan), (B) a plot between fluorescein concentration and normalized voltage output from the portable device, which is linearly dependent on the fluorescence intensity incident on the photodiodes and (C) a comparison between the fluorescence intensity (based on the spectral area) from the commercial fluorometer and the normalized output signal from our portable device.

For the fluorescence measurement, we chose fluorescein, a popular fluorescent dye that is widely used in fluorescent biosensors and chemical sensors [7], [8], [9] as our model sample. We prepared the dye in 1x TE buffer pH 8.0 at various concentrations ranging from 0.000 to 4.000 µM. On the fluorometer, the excitation wavelength was set at 450 nm with 5 nm excitation slit. The emitted fluorescence intensity was summed from 500 nm to 550 nm (in order to match to the measurement range of the developed device) and the results are shown in Fig. 9B. Then the fluorescence intensity from the same set of solutions was measured by our device (from the PD2 output). By comparing the two results obtained from the commercial fluorometer and that from our device, it can be seen that they are highly correlated as shown in Fig. 9C.

**Fig. 10:**
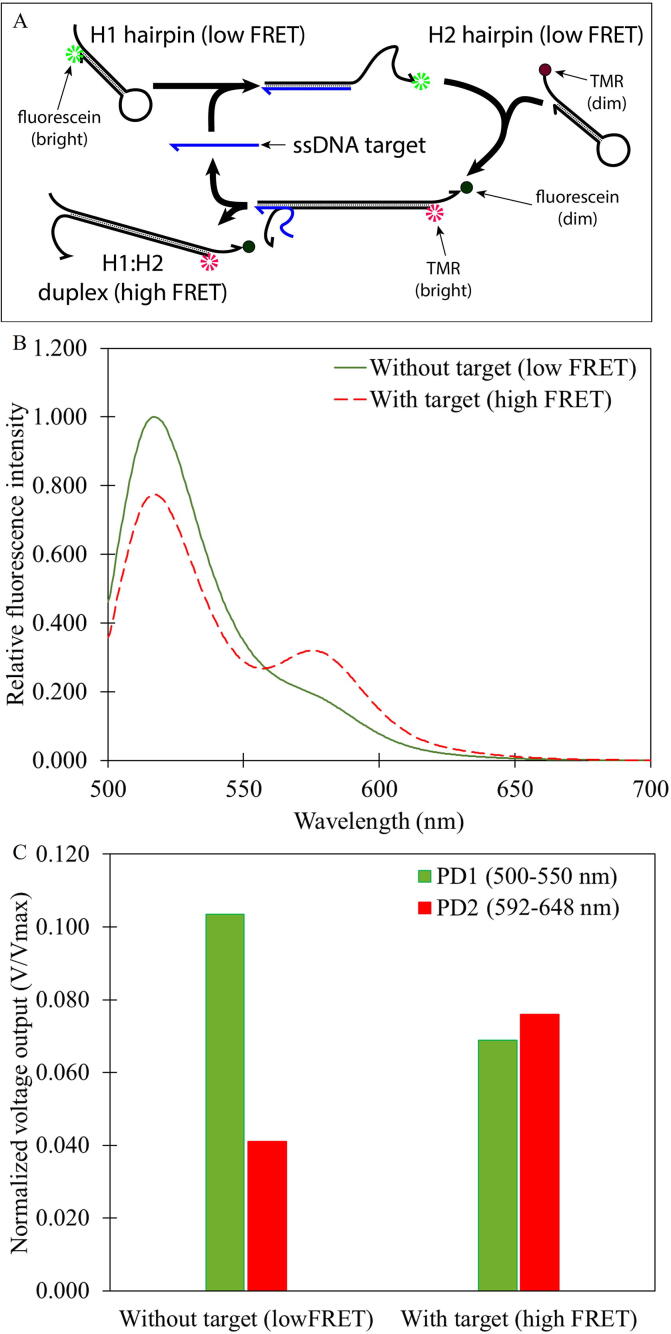
(A) The DNA-based fluorescent sensor based on catalyzed hairpin assembly (CHA) technique being used as the second model sample to test our device. (B) Fluorescence emission spectra from fluorescein and TMR in the absence (solid line) and in the presence (dashed line) of ssDNA target from a commercial fluorometer (F-2700, Hitachi, Japan). (C) Results from our device showing fluorescence intensity measured by the green channel (500–550 nm on PD2) and by the red channel (592–648 nm on PD3).

To demonstrate the full capacity of our device, a DNA based fluorescent sensor doubly labeled with fluorescein and TMR was chosen as the second model sample. The sensor comprises two DNA hairpins, named H1 and H2, as shown in Fig. 10A. They were designed to be in a metastable state and will not interact with each other. However, the right single stranded DNA target will catalyze the H1:H2 duplex formation via the catalyzed hairpin assembly (CHA) reaction [10]. Therefore, the presence of the target ssDNA even at a small amount could result in a large formation of H1:H2 duplex that is easy to detect by, e.g., measuring the Förster resonance energy transfer (FRET) between two fluorophores being labeled on H1 and H2. This FRET based DNA sensor is very sensitive and is found to be widely used in various applications [11], [12], [13]. However, most of the work still rely on commercial fluorometers to measure FRET. In this work, we demonstrate that our device is also capable of measuring FRET. We label H1 with fluorescein (a FRET donor) and H2 with TMR (a FRET acceptor), as shown in Fig. 10A. It is expected that in the absence of target ssDNA, the signal from the green channel (PD2) is high (low FRET when the dyes are well separated) while that on the red channel (PD3) is low (high FRET when the dyes are in close proximity) and vice versa once the ssDNA is introduced. Preliminary results from the commercial fluorometer (Fig. 10B) show that the fluorescence emission from fluorescein (peaked at around 520 nm) is high while that from TMR (peaked at around 578 nm) is low in the absence of the target ssDNA (i.e., low FRET). The opposite is true in the presence of the target ssDNA. Interestingly, our device also recorded the same events as shown in Fig. 10C.

In conclusion, the results from Section 7.1 and 7.2 indicate that our portable device is capable of measuring both light absorption and fluorescence emission from model samples that are widely used in biosensor and chemical sensor development. When compared with commercial equipment (Table 3, Table 4), our device offers a smaller, lighter and cheaper alternative with less power consumption.Therefore, it is in our best interest that our device can be beneficial to researchers whose works require a portable and economical device to detect light absorption or fluorescence emission from optical biosensors or chemical sensors.

**Table 3:** Comparison between a commercial fluorometer (Hitachi F-2700) and the developed device.

**Properties**	Commercial fluorometer(Hitachi F-2700)	The developed device
Excitation wavelength	220–730 nm	450 nm*
Emission wavelength	220–730 nm	500–550 nm* and 592–648 nm*
Light source	150 W Xenon lamp	100 mW* laser diode
Measurable value range	−9999 to 9999	0 to 2048*
Power consumption	400 VA	60 VA
Dimension (W × D × H)	900 × 503 × 343 mm	317 × 224 × 121 mm
Weight	∼ 41 kg	∼ 1 kg
Cost	∼ $ 15,000	∼ $ 810

* Customizable.

**Table 4:** Comparison between Commercial UV–Vis spectrophotometer (Hitachi U-2900) and the developed device.

**Properties**	Commercial UV–Vis spectrophotometer (Hitachi U-2900)	The developed device
Wavelength	190 to 1100 nm	450 nm*
Light source	Tungsten and deuterium lamps	100 mW* laser diode
Measurable value range	−3 to 3	0.00 to 1.00*
Power consumption	300 VA	60 VA
Dimension (W × D × H)	500 × 605 × 283 mm	317 × 224 × 121 mm
Weight	∼ 31 kg	∼ 1 kg
Cost	∼ $ 6,000	∼ $ 810

* Customizable.

## CRediT authorship contribution statement

**Kittirat Phooplub:** Conceptualization, Methodology, Software, Investigation, Validation, Visualization, Writing – original draft, Writing – review & editing. **Sirirat Ouiganon:** Validation. **Panote Thavarungkul:** Supervision. **Proespichaya Kanatharana:** Supervision. **Chittanon Buranachai:** Supervision, Conceptualization, Methodology, Visualization, Funding acquisition, Resources, Project administration, Writing – review & editing.

## Declaration of Competing Interest

The authors declare that they have no known competing financial interests or personal relationships that could have appeared to influence the work reported in this paper.
