# Supplementary material for: Portable device for dual detection of fluorescence and absorbance for biosensing or chemical sensing applications
Source: HardwareX. 2022 Jan 28;11:e00268. doi: 10.1016/j.ohx.2022.e00268 (PMC9058716; doi:10.1016/j.ohx.2022.e00268)
Supplement: Supplementary data 1 [file mmc1.pdf]

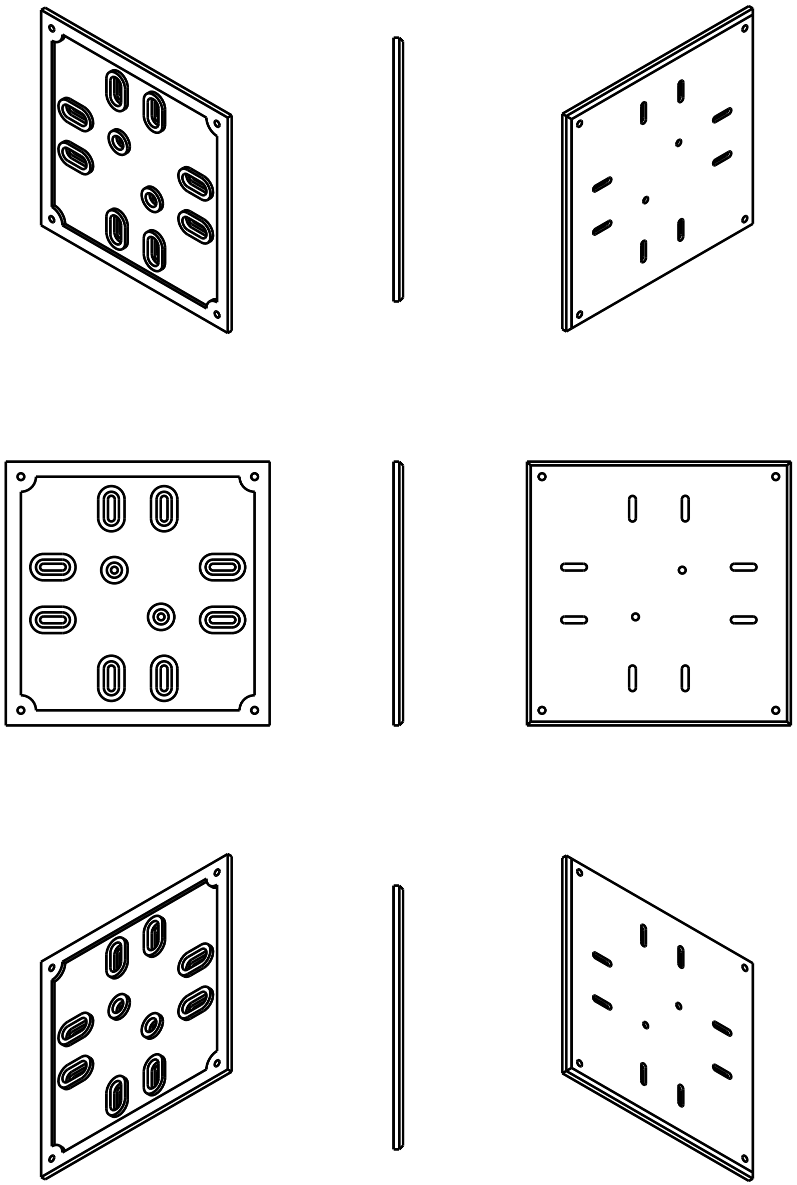

DESIGNED BY:
Phooplub, K., et.al.
DATE:
13/10/2021

Title Part 1

Subtitle

SIZE
A4

SCALE	WEIGHT (kg)	DRAWING NUMBER	SHEET
Scale	Weight	1	1

G	_
F	_
E	_
D	_
C	_
B	_
A	_

This drawing is our property; it can't be reproduced or communicated without our written consent.

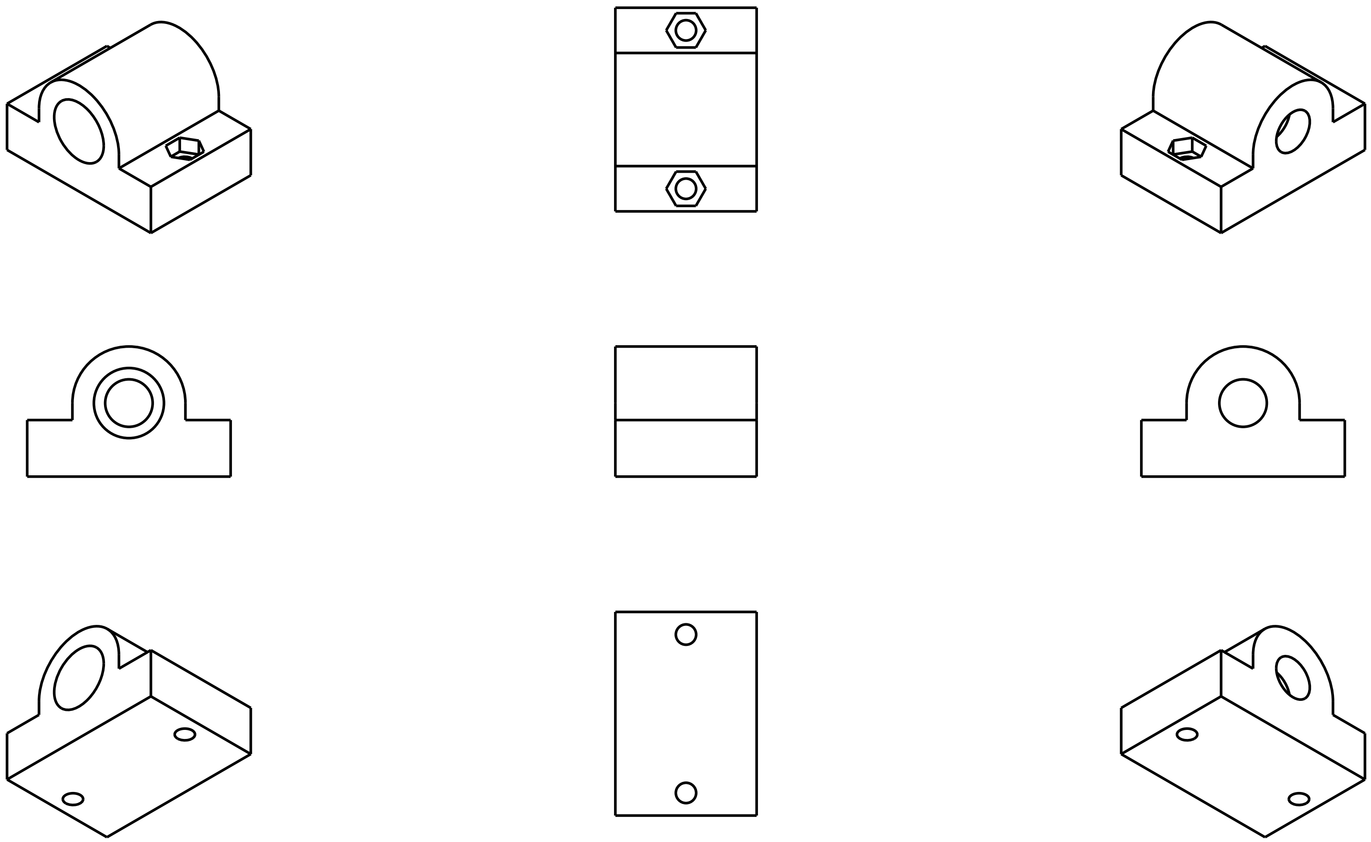

DESIGNED BY:
Phooplub, K., et.al.
DATE:
13/10/2021

Title Part 2

Subtitle

SIZE	
A4	

SCALE	WEIGHT (kg)	DRAWING NUMBER	SHEET
Scale	Weight	2	2

G	_
F	_
E	_
D	_
C	_
B	_
A	_

This drawing is our property; it can't be reproduced or communicated without our written consent.

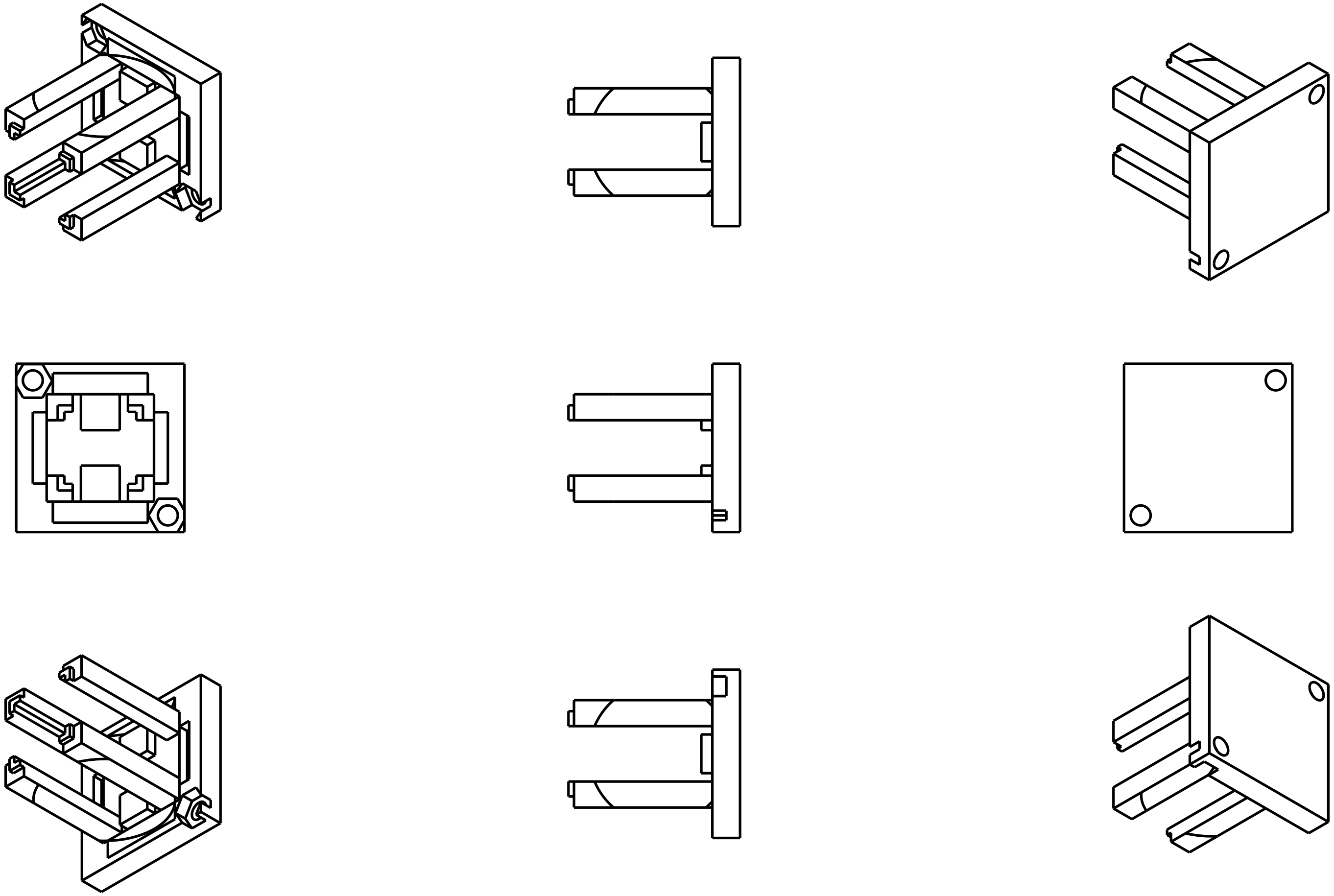

DESIGNED BY: Phooplub, K., et.al. DATE: 13/10/2021	Title Part 3 Subtitle		
SIZE A4			
SCALE Scale	WEIGHT (kg) Weight	DRAWING NUMBER 3	SHEET 3

This drawing is our property; it can't be reproduced or communicated without our written consent.

G	_
F	_
E	_
D	_
C	_
B	_
A	_

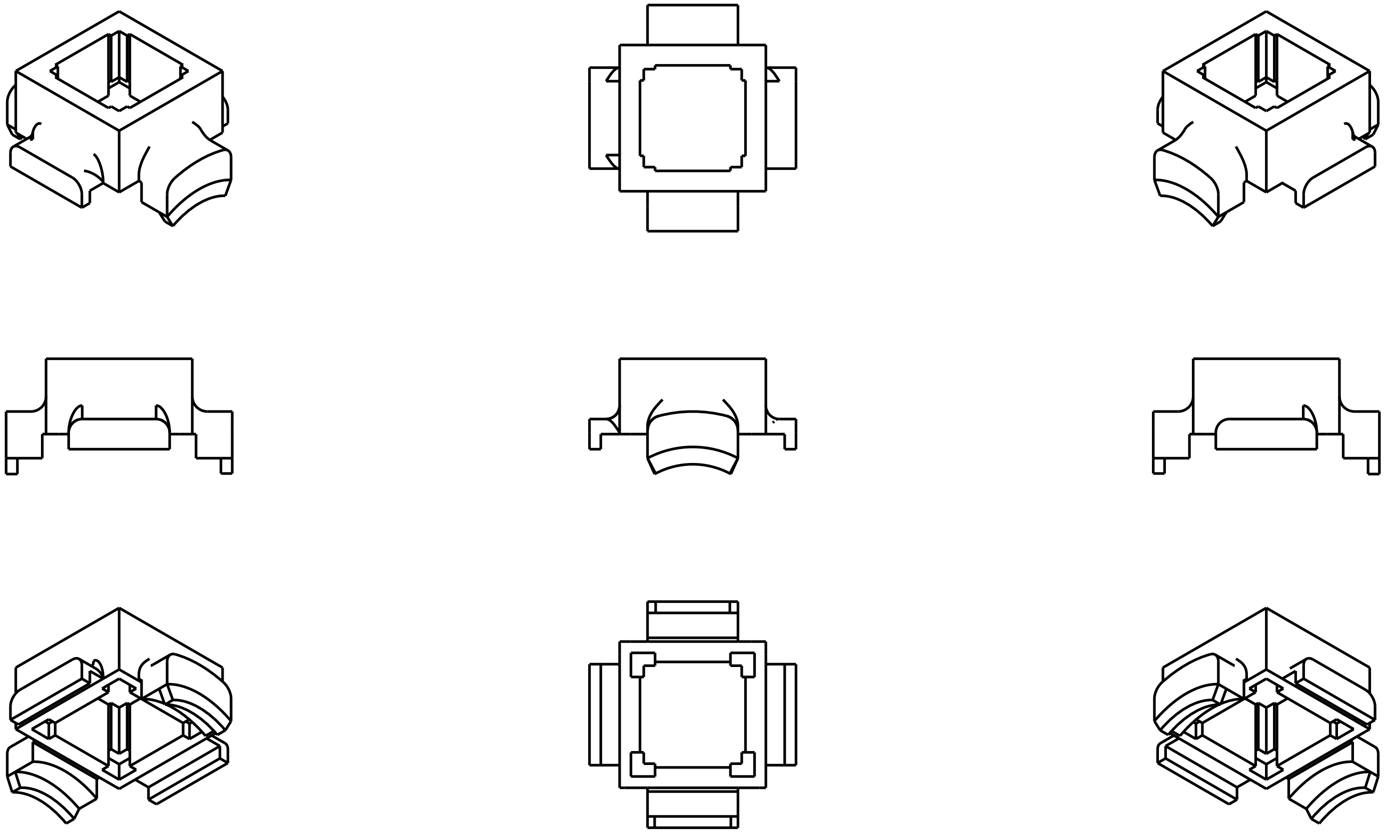

DESIGNED BY:
Phooplub, K., et.al.
DATE:
13/10/2021

Title Part 4

Subtitle

SIZE
A4

SCALE	WEIGHT (kg)	DRAWING NUMBER	SHEET
Scale	Weight	4	4

This drawing is our property; it can't be reproduced or communicated without our written consent.

G	_
F	_
E	_
D	_
C	_
B	_
A	_

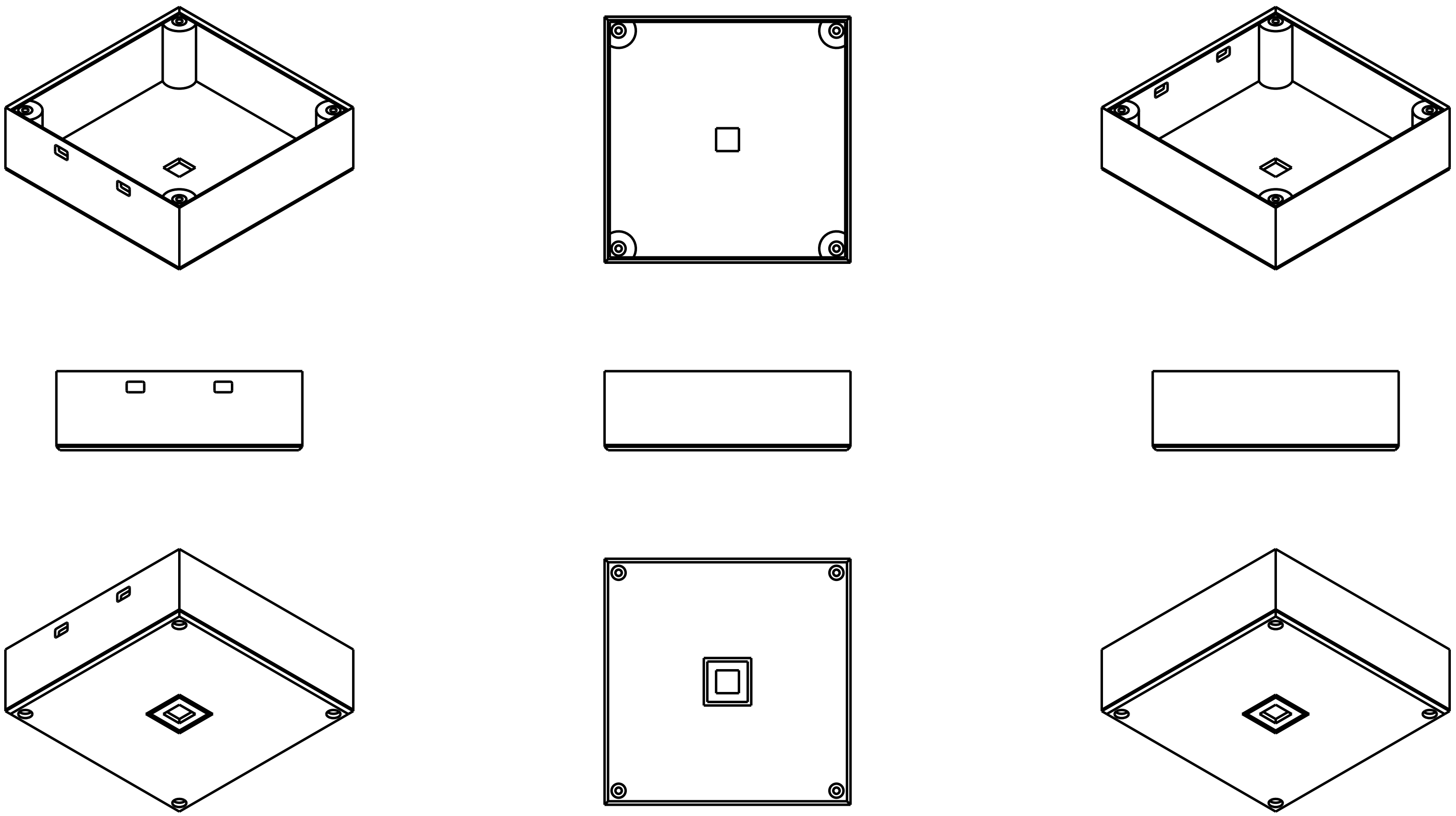

DESIGNED BY:
Phooplub, K., et.al.
DATE:
13/10/2021

Title Part 5

Subtitle

SIZE	SCALE	WEIGHT (kg)	DRAWING NUMBER	SHEET
A4	Scale	Weight	5	5

G	_
F	_
E	_
D	_
C	_
B	_
A	_

This drawing is our property; it can't be reproduced or communicated without our written consent.

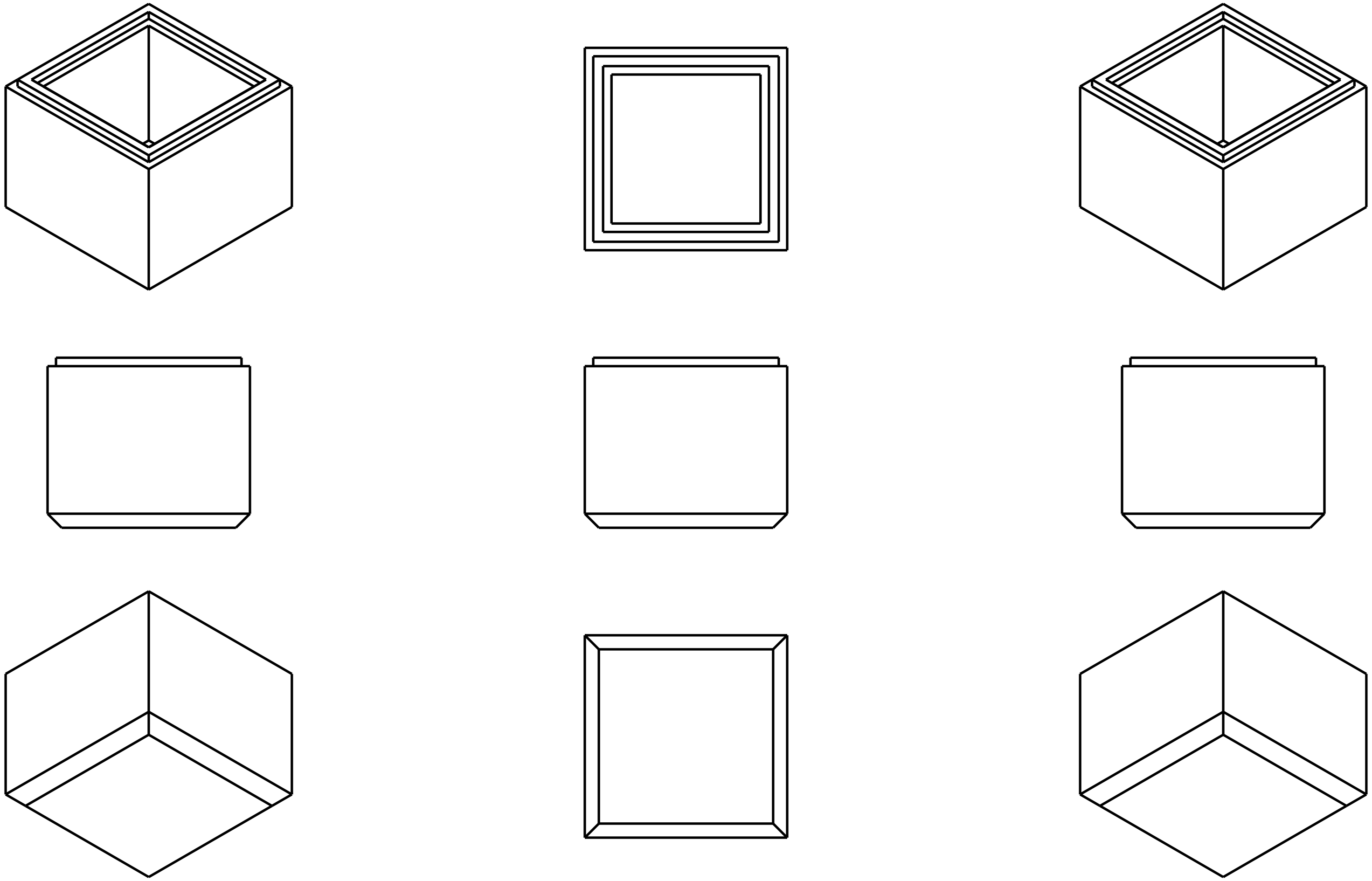

DESIGNED BY: Phooplub, K., et.al. DATE: 13/10/2021		Title Part 6 Subtitle	
SIZE A4			
SCALE Scale	WEIGHT (kg) Weight	DRAWING NUMBER 6	SHEET 6

This drawing is our property; it can't be reproduced or communicated without our written consent.

G	_
F	_
E	_
D	_
C	_
B	_
A	_

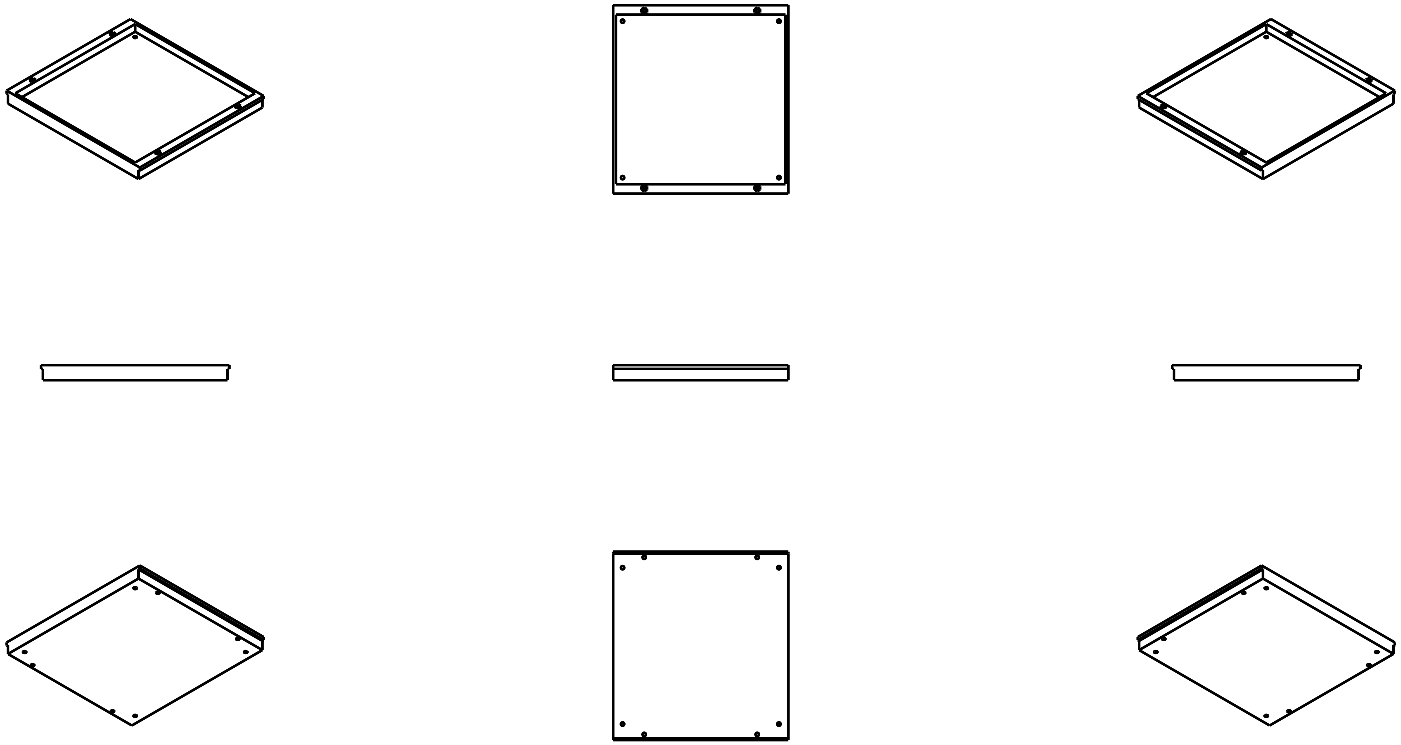

DESIGNED BY: Phooplub, K., et.al. DATE: 13/10/2021		Title Part 7 Subtitle	
SIZE A4			
SCALE Scale	WEIGHT (kg) Weight	DRAWING NUMBER 7	SHEET 7

This drawing is our property; it can't be reproduced or communicated without our written consent.

G	_
F	_
E	_
D	_
C	_
B	_
A	_

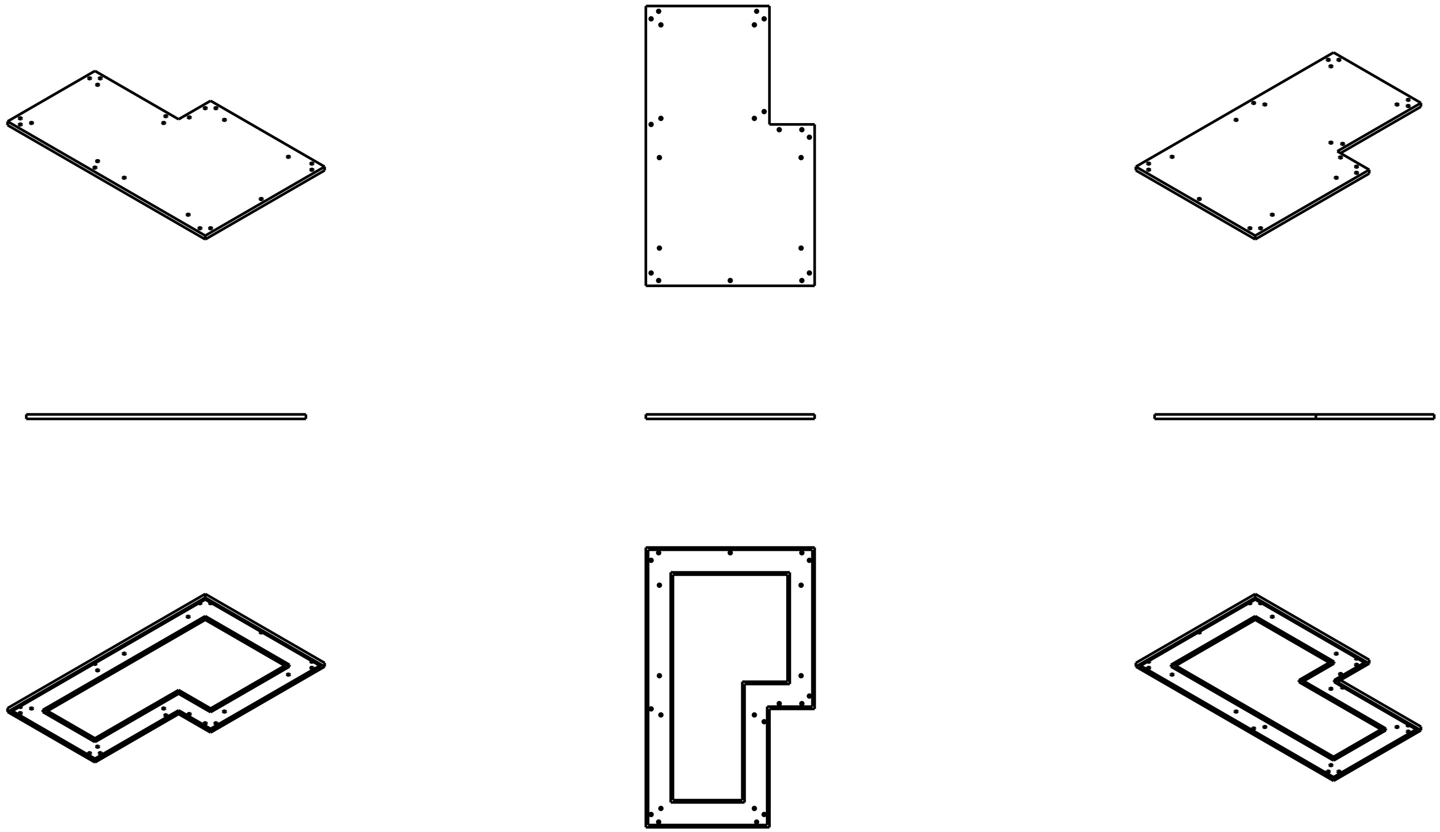

DESIGNED BY: Phooplub, K., et.al. DATE: 13/10/2021	Title Part 8 Subtitle		
SIZE A4			
SCALE Scale	WEIGHT (kg) Weight	DRAWING NUMBER 8	SHEET 8

G	_
F	_
E	_
D	_
C	_
B	_
A	_

This drawing is our property; it can't be reproduced or communicated without our written consent.

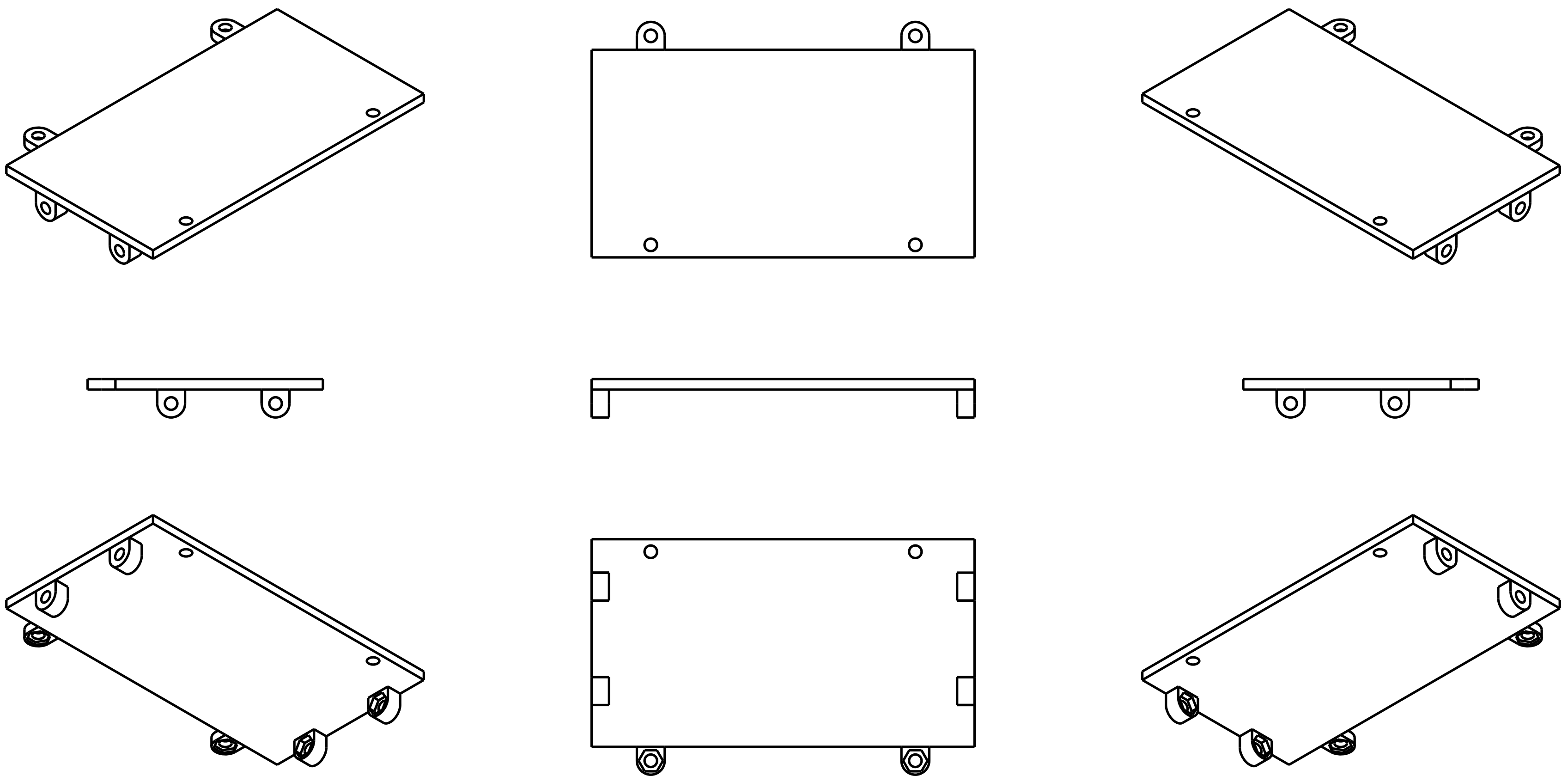

DESIGNED BY:
Phooplub, K., et.al.
DATE:
13/10/2021

Title Part 9

Subtitle

SIZE	
A4	

SCALE	WEIGHT (kg)	DRAWING NUMBER	SHEET
Scale	Weight	9	9

G	_
F	_
E	_
D	_
C	_
B	_
A	_

This drawing is our property; it can't be reproduced or communicated without our written consent.

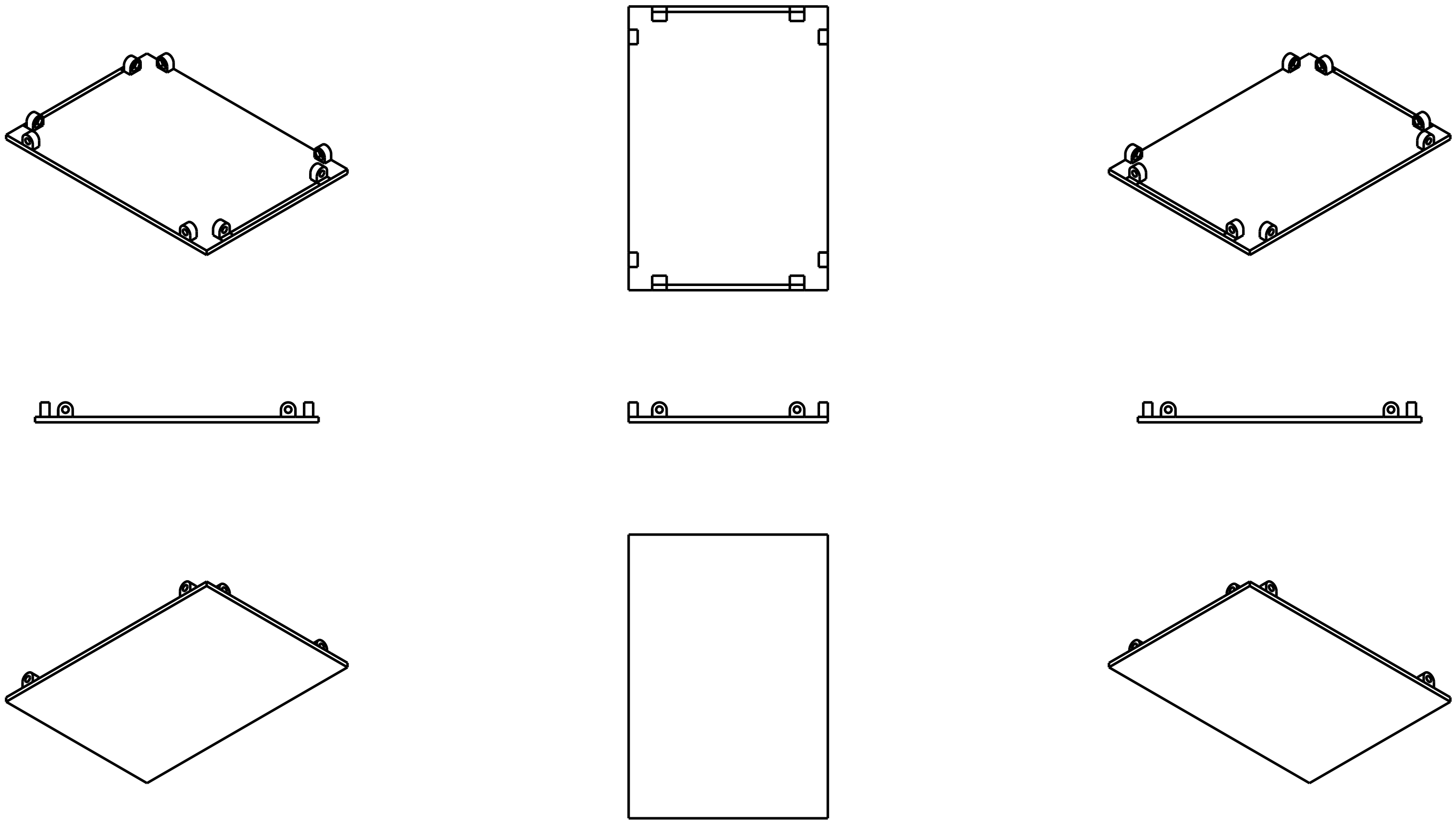

DESIGNED BY:
Phooplub, K., et.al.
DATE:
13/10/2021

Title Part 10

Subtitle

SIZE		
A4		

SCALE	WEIGHT (kg)	DRAWING NUMBER	SHEET
Scale	Weight	10	10

G	_
F	_
E	_
D	_
C	_
B	_
A	_

This drawing is our property; it can't be reproduced or communicated without our written consent.

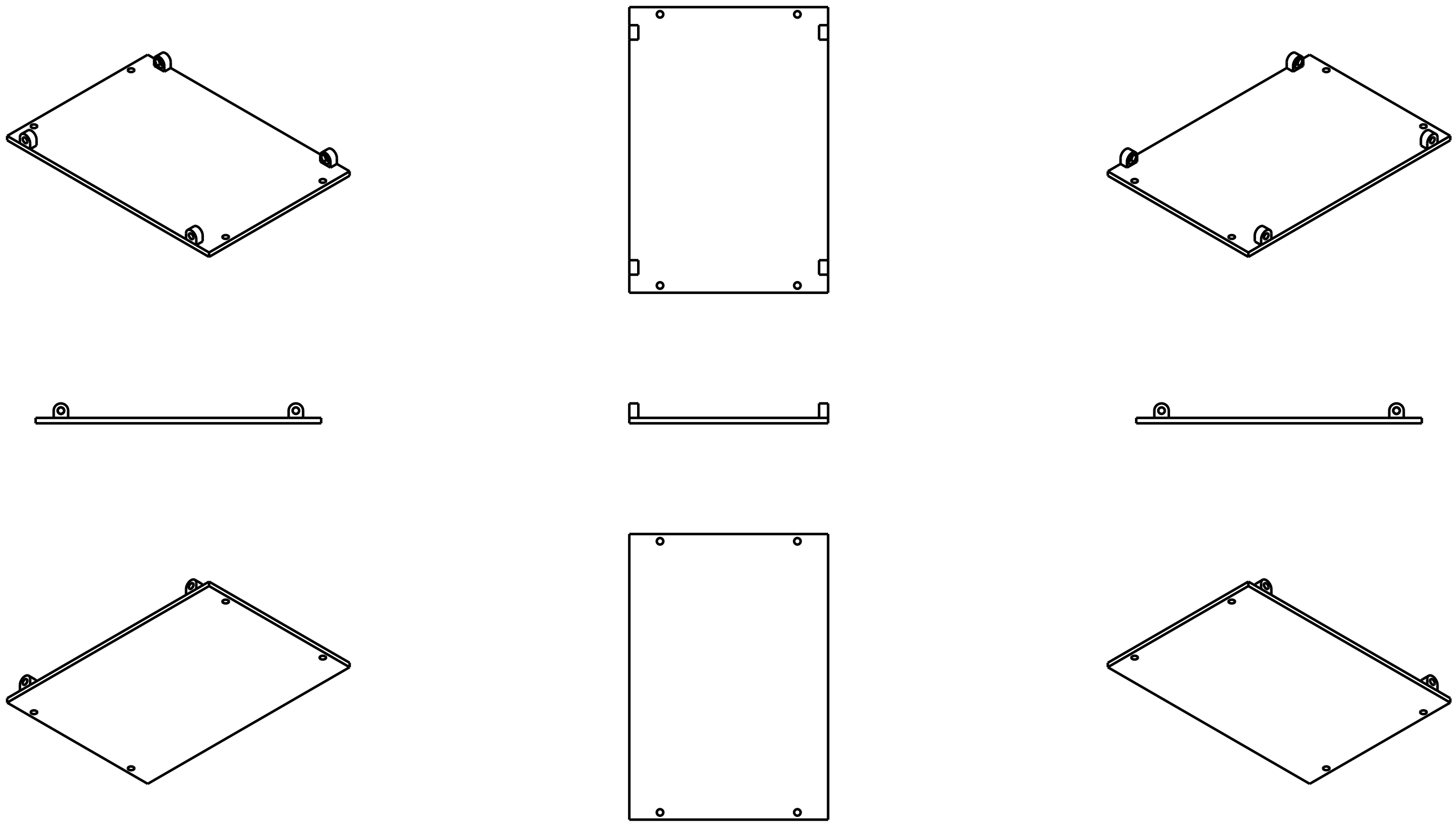

DESIGNED BY: Phooplub, K., et.al. DATE: 13/10/2021		Title Part 11 Subtitle	
SIZE A4			
SCALE Scale	WEIGHT (kg) Weight	DRAWING NUMBER 11	SHEET 11

This drawing is our property; it can't be reproduced or communicated without our written consent.

G	_
F	_
E	_
D	_
C	_
B	_
A	_

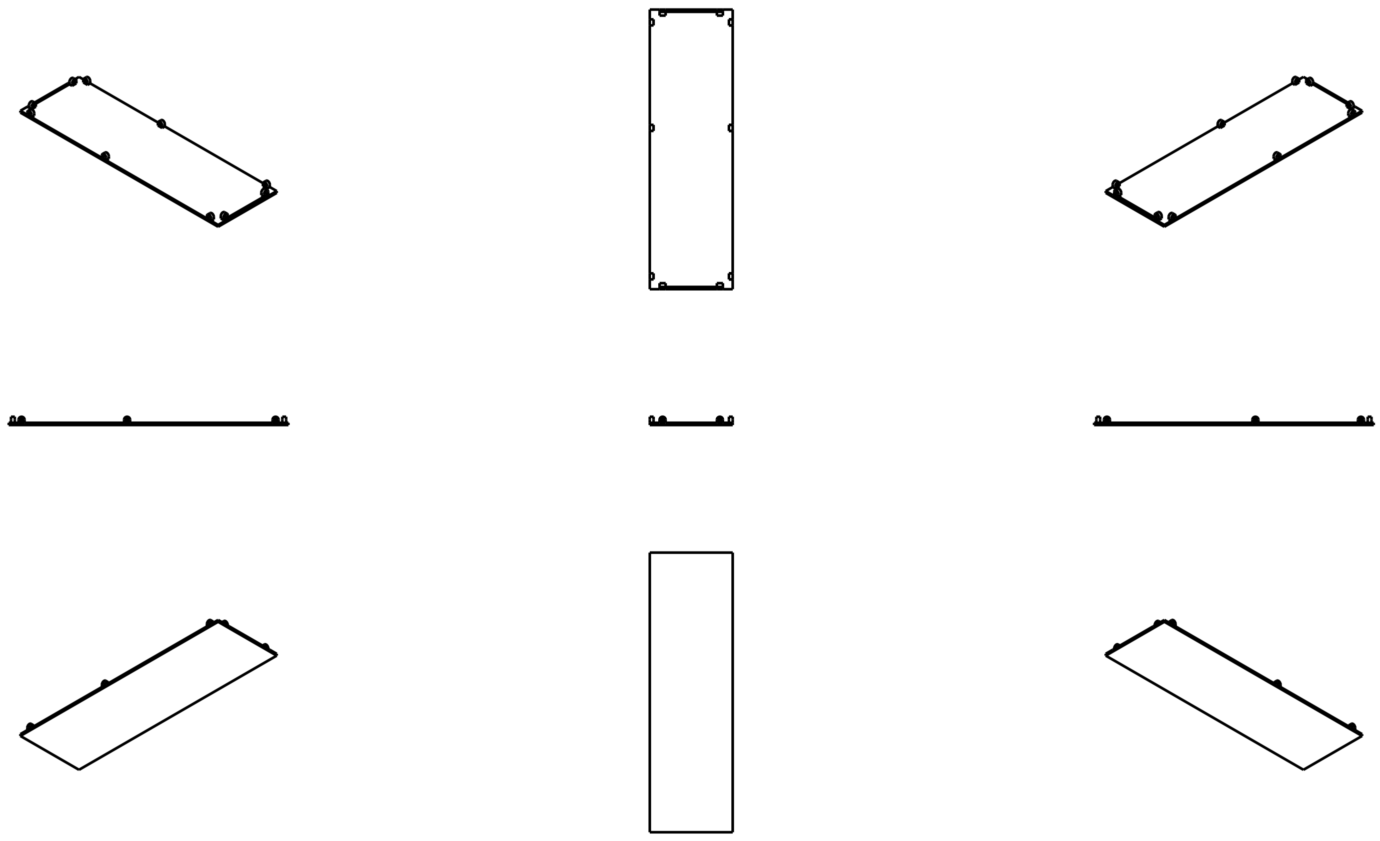

DESIGNED BY: Phooplub, K., et.al. DATE: 13/10/2021		Title Part 13 Subtitle	
SIZE A4			
SCALE Scale	WEIGHT (kg) Weight	DRAWING NUMBER 12	SHEET 12

This drawing is our property; it can't be reproduced or communicated without our written consent.

G	_
F	_
E	_
D	_
C	_
B	_
A	_

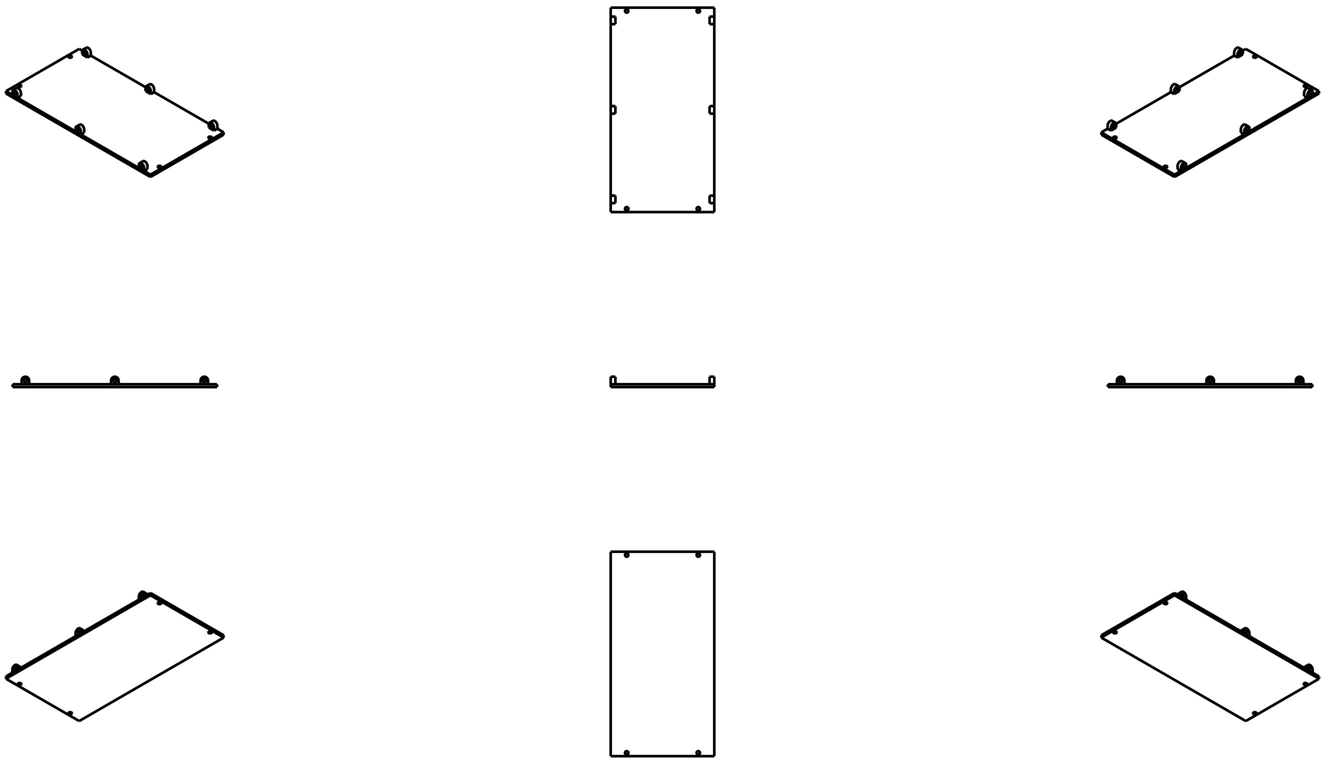

DESIGNED BY:
Phooplub, K., et.al.

DATE:
13/10/2021

Title Part 13

Subtitle

SIZE			
A4			
SCALE	WEIGHT (kg)	DRAWING NUMBER	SHEET
Scale	Weight	13	13

This drawing is our property; it can't be reproduced or communicated without our written consent.

G	_
F	_
E	_
D	_
C	_
B	_
A	_

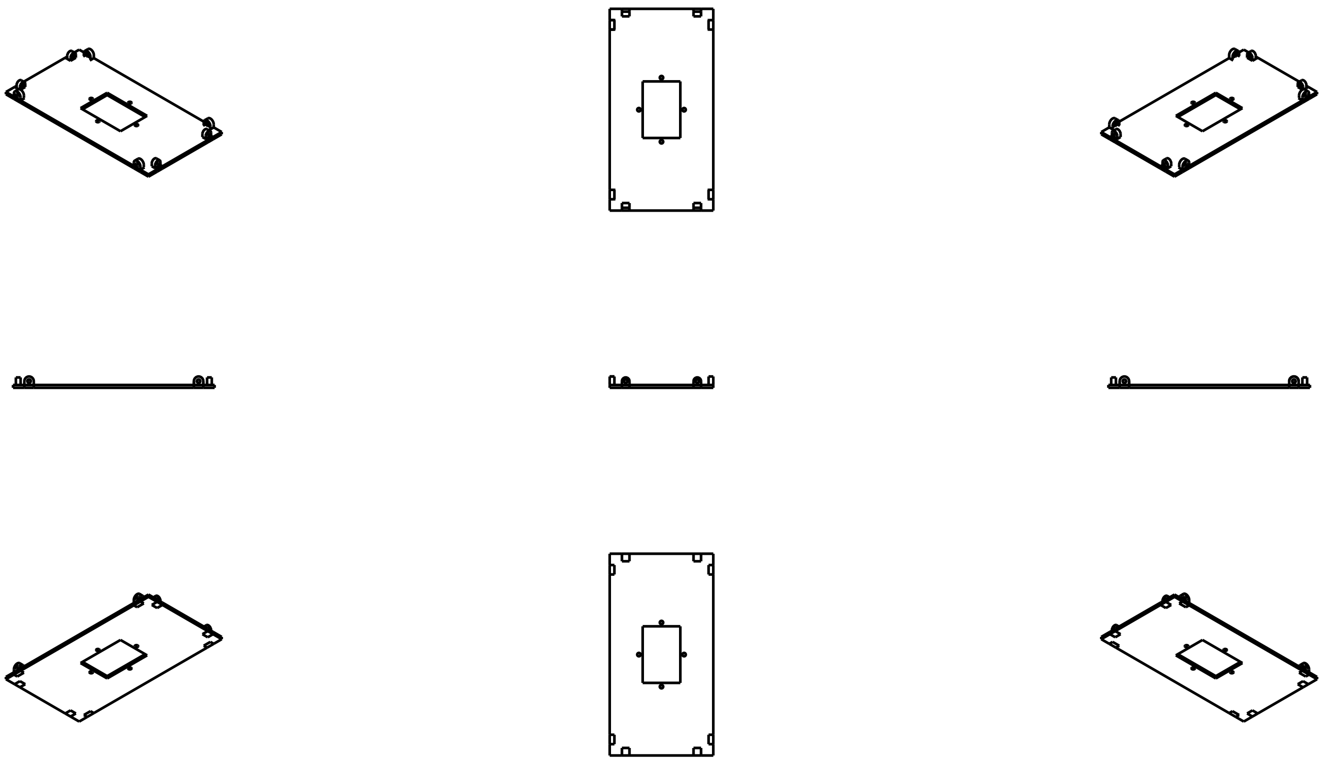

DESIGNED BY:
Phooplub, K., et.al.
DATE:
13/10/2021

Title Part 14

Subtitle

SIZE		
A4		

SCALE	WEIGHT (kg)	DRAWING NUMBER	SHEET
Scale	Weight	14	14

This drawing is our property; it can't be reproduced or communicated without our written consent.

G	_
F	_
E	_
D	_
C	_
B	_
A	_

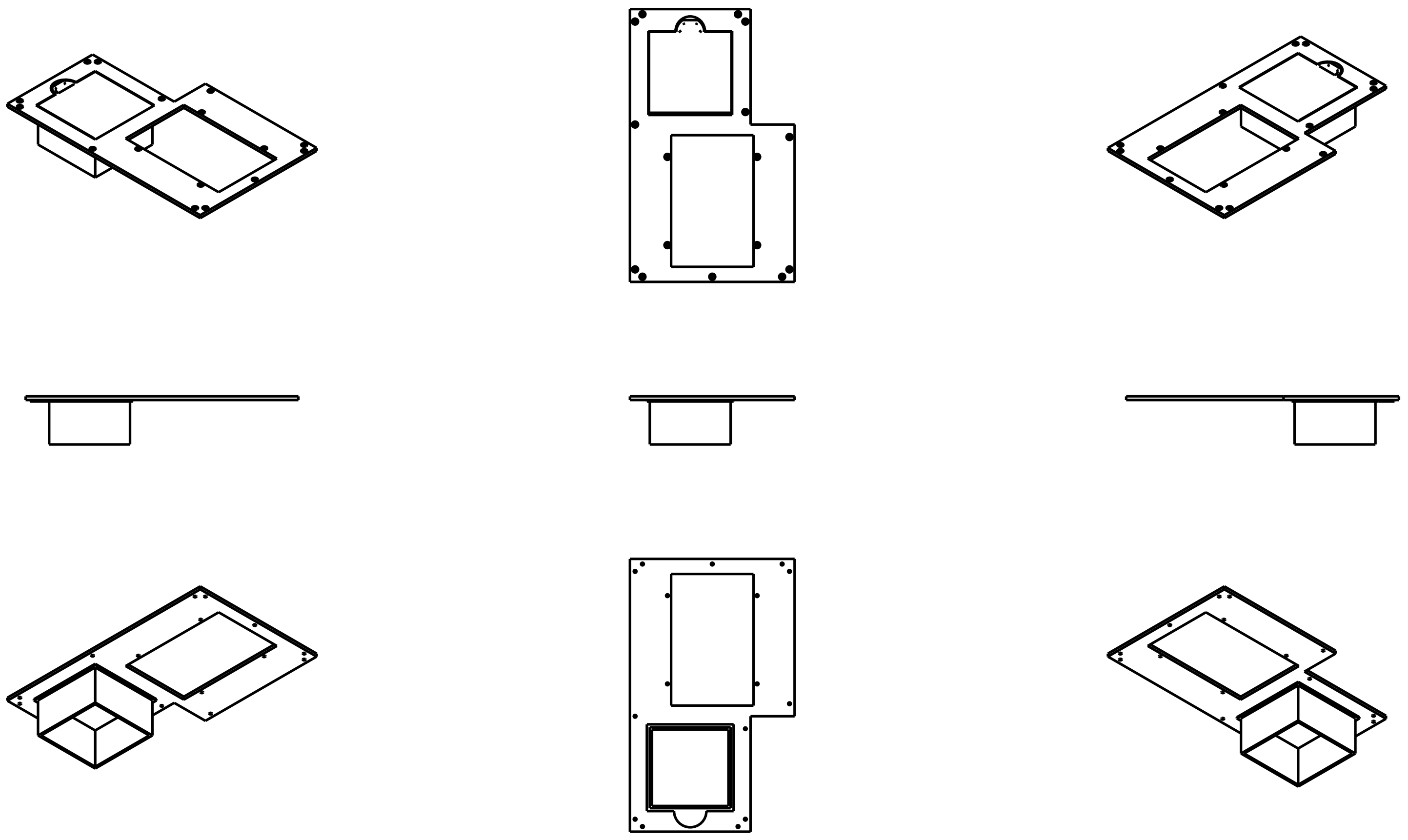

DESIGNED BY: Phooplub, K., et.al. DATE: 13/10/2021		Title Part 15 Subtitle		G	_
				F	_
SIZE A4				E	_
				D	_
SCALE Scale	WEIGHT (kg) Weight	DRAWING NUMBER 15	SHEET 15	C	_
				B	_
				A	_

This drawing is our property; it can't be reproduced or communicated without our written consent.

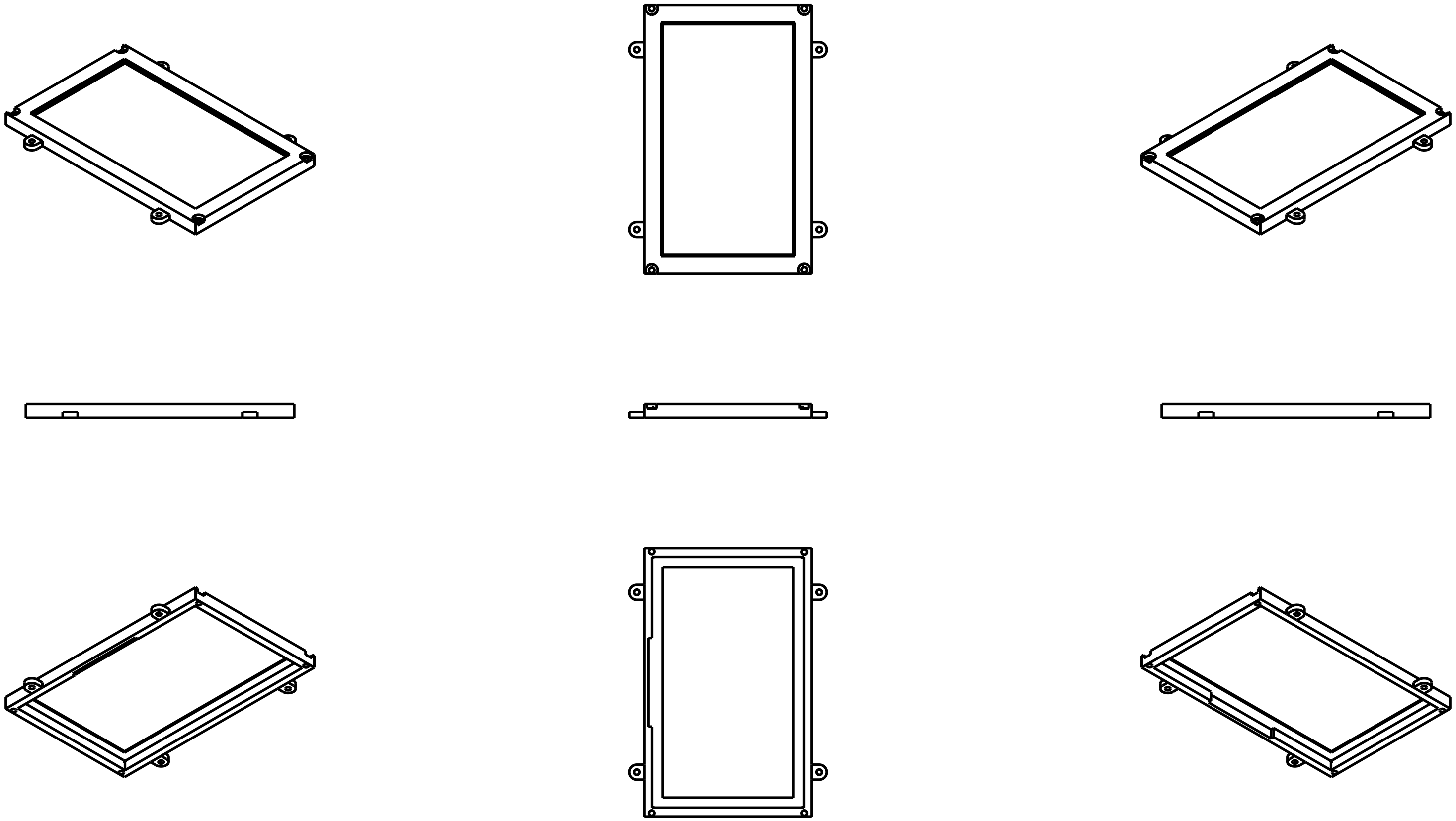

DESIGNED BY:
Phooplub, K., et.al.
DATE:
13/10/2021

Title Part 16

Subtitle

SIZE A4			
SCALE Scale	WEIGHT (kg) Weight	DRAWING NUMBER 16	SHEET 16

This drawing is our property; it can't be reproduced or communicated without our written consent.

G	_
F	_
E	_
D	_
C	_
B	_
A	_

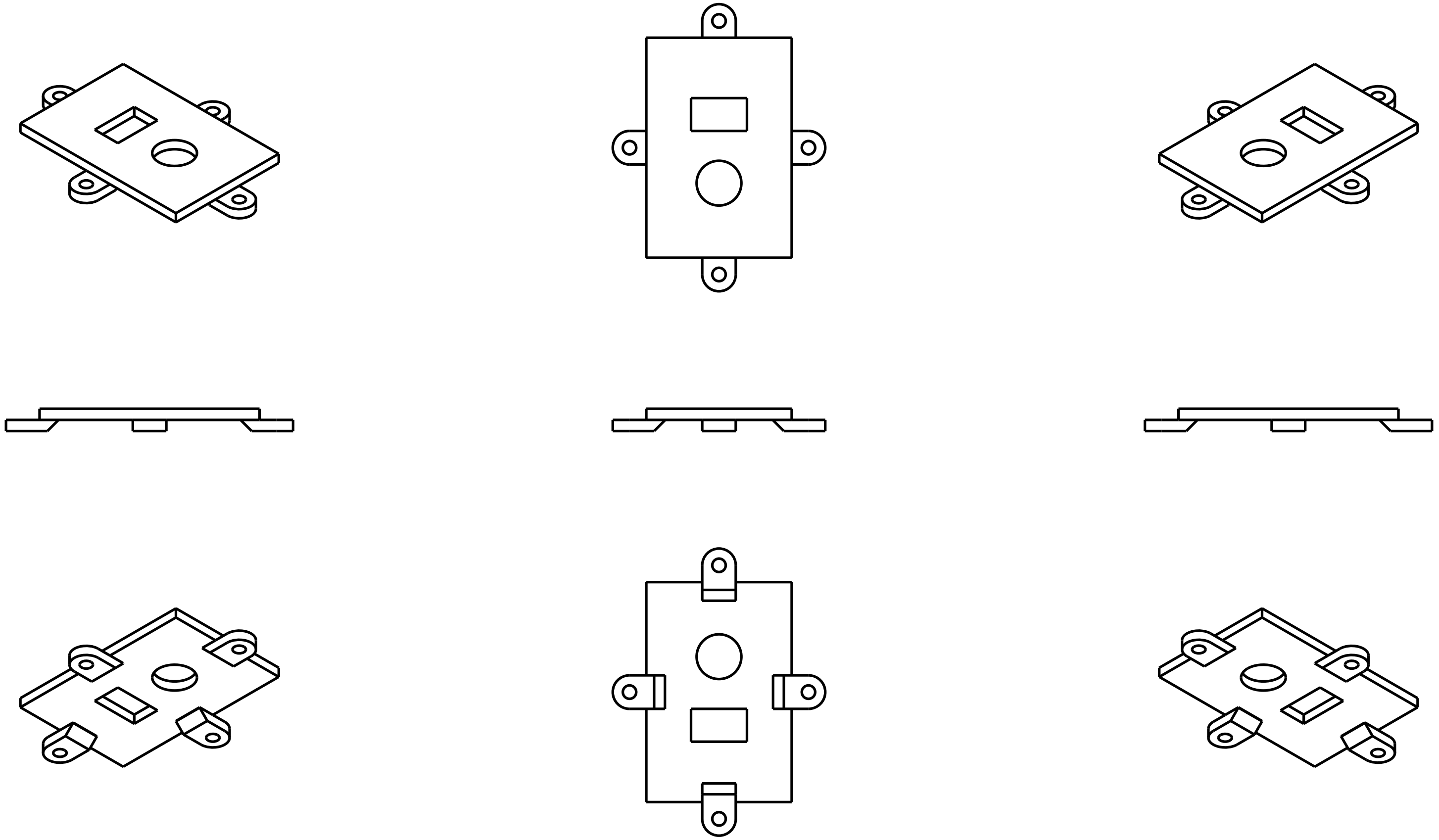

DESIGNED BY: Phooplub, K., et.al. DATE: 13/10/2021	Title Part 17 Subtitle		
SIZE A4			
SCALE Scale	WEIGHT (kg) Weight	DRAWING NUMBER 17	SHEET 17

This drawing is our property; it can't be reproduced or communicated without our written consent.

G	_
F	_
E	_
D	_
C	_
B	_
A	_
